# Monitoring Vaccine-Induced Antibody Levels Using Carbon Nanotube-Based Field-Effect Transistors

**DOI:** 10.1021/acs.analchem.5c03817

**Published:** 2025-11-03

**Authors:** Amir Amiri, Wenting Shao, Zidao Zeng, Ashish Dhayani, Stephen C. Balmert, Louis D. Falo, Emrullah Korkmaz, Alexander Star

**Affiliations:** † Department of Chemistry, 6614 University of Pittsburgh, Pittsburgh, Pennsylvania 15260, United States; ‡ Department of Dermatology, 12317 University of Pittsburgh School of Medicine, 200 Lothrop Street, Biomedical Science Tower, Pittsburgh, Pennsylvania 15213, United States; § Department of Bioengineering, University of Pittsburgh, Pittsburgh, Pennsylvania 15261, United States; ∥ Clinical and Translational Science Institute, University of Pittsburgh, Pittsburgh, Pennsylvania 15213, United States; ⊥ The McGowan Institute for Regenerative Medicine, University of Pittsburgh, Pittsburgh, Pennsylvania 15219, United States

## Abstract

Vaccines are powerful public health tools to protect against emerging infectious pathogens. Multiple vaccine doses are typically required to achieve robust protection that is often mediated by the induction of pathogen-specific antibodies. Thus, monitoring the levels of vaccine-induced antibodies in immunized individuals is crucial to ensuring vaccine effectiveness and compliance. However, existing antibody-detection techniques are resource- and time-inefficient, highlighting the need for improved technologies for monitoring vaccine-induced antibody levels. Here, we developed a field-effect transistor (FET) biosensor platform based on antigen-functionalized semiconducting single-walled carbon nanotubes (SWCNTs) for the rapid and convenient detection of pathogen-specific antibodies. Our antibody sensor platform was designed to produce robust signals with a high signal-to-noise ratio upon antigen–antibody interactions altering the electrical conductivity of interconnected SWCNTs. Key physicoelectrochemical characteristics of our SWCNT FET biosensor were validated by atomic force microscopy (AFM), scanning electron microscopy (SEM), Raman spectroscopy, and FET measurements. Robust and rapid antibody detection capability of our SWCNT FET biosensor platform was demonstrated by measuring virus-specific antibodies (e.g., anti-hemagglutinin (anti-HA), anti-SARS-CoV-2 nucleocapsid (anti-N), and anti-SARS-CoV-2 spike (anti-S) antibodies) in different systems. Our nanoelectronic sensor platform was able to detect these antibodies in a wide linear concentration range of 100 ag/mL to 100 ng/mL. Owing to the direct attachment of the corresponding antigens to SWCNTs, desirable limits of detection of 0.20 and 20.6 ag/mL were obtained for the detection of anti-HA and anti-S antibodies, respectively. Together, our SWCNT FET biosensor platform offers a next-generation antibody detection technology capable of low-cost, rapid, accessible, and convenient monitoring of vaccine-induced antibodies.

## Graphical abstract

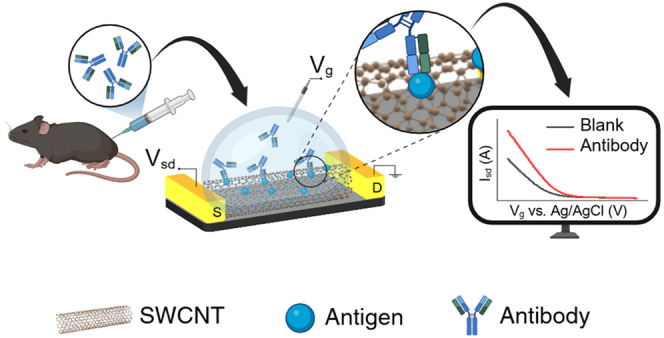

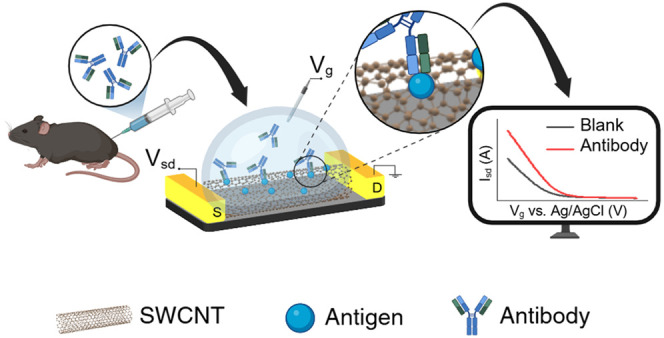

## Introduction

The annual outbreaks of influenza viruses and coronaviruses during the winter season pose a great threat to public health.^1–3^ Hemagglutinin (HA) and neuraminidase (NA) glycoproteins on the surface of influenza A subtype virus H1N1 play crucial roles in binding to host cell sialic acid (SA) receptors for cell entry and replication.^4^ The influenza virus infects 3–5 million people worldwide annually, resulting in 290,000 to 650,000 deaths.^5^ The severe acute respiratory syndrome coronavirus 2 (SARS-CoV-2) causes the coronavirus disease 2019 (COVID-19).^6^ A large positive-stranded RNA genome in SARS-CoV-2 encodes four structural proteins: spike (S), envelope (E), matrix (M), and nucleocapsid (N).^7^ The virus emerged in December 2019, and within six months of the first pandemic wave, it caused over 500,000 deaths.^8^ On April 1, 2025, Johns Hopkins University’s assessment showed that the number of deaths worldwide had surpassed 6 million.^9^

Safe, effective, and durable vaccines offer a viable public health tool to address the significant mortality associated with influenza viruses and coronaviruses.^10^ However, waning vaccine-induced immunity remains a major challenge, highlighting the need for multiple-dose vaccines. Upon vaccination, the host generates orchestrated immune responses to the targeted virus. Protective immunity induced by vaccination is often mediated by the presence of virus-specific antibodies that prevent viral entry into host cells and, in turn, virus replication,^11^ highlighting the importance of frequent monitoring of these antibody levels for personalized immunization regimens.^12^

Several methods are routinely used for the determination of the magnitude and quality of virus-specific antibodies. The hemagglutination inhibition (HI) assay,^11,13^ enzyme-linked immunosorbent assay (ELISA),^14–20^ and western blot (WB) assay^21^ are frequently used for the detection of virus-specific antibodies. However, these commonly used assays for antibody detection are suboptimal for the frequent monitoring of vaccine-induced antibody levels. In addition to being time-consuming and laborious, they often require a large sample volume and expensive equipment.^22^ Further, they are not amenable to miniaturization and cannot be used as portable sensors.^23,24^

Recently, biochemical sensors have attracted increasing attention as effective alternatives to traditional detection assays owing to their simplicity, high selectivity and sensitivity, and compatibility with nanotechnology to be applied as disposable sensors.^25–27^ Specifically, field-effect transistor (FET) biosensors have been widely used for point-of-care diagnosis because of their fast response time, label-free detection, and low detection limit.^28^ A FET biosensor is typically a three-terminal device consisting of source, drain, and gate electrodes with semiconductor channel and biorecognition sites.^22^ The recognition mechanism of FET biosensors is based on the selective interaction of the biomolecule of interest with the specific immobilized receptor on the surface of the biosensor.^29^ The analyte–receptor interactions affect the current between source and drain electrodes by electrostatic gating of the semiconductor channel, enabling signal amplification and transduction of FET biosensors.^30,31^ This biorecognition involves the functional and structural affinity of analyte toward the receptor, providing label-free detection by FET biosensors.^32^

Carbon nanotubes (CNTs) are among the most promising semiconducting materials for biomolecular detection and FET biosensor fabrication.^33–36^ CNTs have numerous advantages, including small diameter, high aspect ratio, large surface area, excellent mechanical strength, and high conductivity.^37,38^ Based on the number of walls, CNTs can be classified as single-walled carbon nanotubes (SWCNTs) and multiwalled carbon nanotubes (MWCNTs).^39^ As the main biosensing mechanism is to measure the electric changes of the semiconductor toward the analyte–receptor interactions, SWCNTs demonstrated greater potential than MWCNTs to fabricate highly sensitive FET devices because all of their atoms are on the surface.^40^ Their preferred high intrinsic carrier mobility and low charge-carrier density facilitate charge transfer during biosensing, leading to the detection of electrostatic interactions.^41^ A key step in the fabrication of FET biosensors is providing a convenient surface for the immobilization of either an antibody or antigen as receptors. To ensure stable immobilization of bioreceptors on SWCNTs, 1-ethyl-3-(3-(dimethylamino)­propyl) carbodiimide/*N*-hydroxysulfosuccinimide (EDC/sulfo-NHS) coupling is a widely used approach for creating reactive groups on the surface. The interactions between amine or carboxylic acid functional groups in the structure of biomolecular receptors (e.g., antibodies) and chemically attached reactive functional groups onto SWCNTs secure stable attachment of receptors.^42,43^

In this work, we demonstrate the development of FET biosensors for the selective detection of influenza-virus- and coronavirus-specific antibodies. The FET biosensors were fabricated through the functionalization of SWCNTs with influenza A H1N1 hemagglutinin (HA), SARS-CoV-2 spike, or SARS-CoV-2 nucleocapsid proteins to achieve selective detection of anti-hemagglutinin (anti-HA), anti-SARS-CoV-2 S (anti-S), or anti-SARS-CoV-2 N (anti-N) proteins, respectively. Under optimized conditions, the designed HA-functionalized SWCNT (HA-SWCNT), SARS-CoV-2 spike-functionalized SWCNT (S-SWCNT), and SARS-CoV-2 nucleocapsid-functionalized SWCNT (N-SWCNT) FET biosensors were applied to detect anti-HA, anti-S and anti-N antibodies, respectively, within a wide concentration range of 100 ag/mL to 10 μg/mL in phosphate-buffered saline (PBS) and artificial interstitial fluid (ISF). Rapid detection of anti-HA in artificial ISF by the HA-SWCNT FET biosensor highlights the potential of this approach for application as wearable biosensors for on-site detection. The proposed approach can ultimately facilitate monitoring vaccination effectiveness by detecting vaccine-induced antibody levels over time.

## Experimental Section

### Materials

Semiconductor-enriched SWCNTs (IsoSol-S100 polymer-wrapped nanotubes) were obtained from NanoIntegris. Sylgard 184 silicone elastomer base and curing agent were purchased from the Dow company. Sodium chloride, sodium bicarbonate, magnesium sulfate anhydrous, Pierce premium-grade 1-ethyl-3-(3-(dimethylamino)­propyl) carbodiimide, *N*-hydroxysulfosuccinimide, polyethylene glycol, and SARS-CoV-2 coronavirus spike protein (subunit 1) polyclonal antibody were purchased from Thermo Fisher Scientific, USA.

Biotinylated SARS-CoV-2 (COVID-19) S protein RBD was purchased from AcroBiosystems. Recombinant coronavirus N protein and anti-SARS-CoV-2 N antibody were purchased from BioVision Inc., USA.

Influenza A H1N1 (A/California/04/2009) hemagglutinin/HA protein (ECD-His Tag), Influenza A H1N1 (Swine Flu 2009) hemagglutinin/HA mouse monoclonal antibody, and SARS-CoV2 (2019-nCoV) Spike S1 + S2 (ECD-His Tag) Recombinant Protein were purchased from Sino Biological, USA.

A Thermo Scientific Barnstead Nanopure system with resistivity >18.2 MΩ·cm was used to provide nanopure water. Calcium chloride, sodium phosphate monobasic dihydrate, potassium chloride, d-(+)-glucose, sodium gluconate, Tween 20, and phosphate buffer saline tablet were purchased from Sigma-Aldrich, USA. Artificial interstitial fluid (ISF) was prepared as follows: 6.3 g/L NaCl, 0.26 g/L KCl, 0.17 g/L CaCl_2_, 0.17 g/L MgSO_4_, 2.2 g/L NaHCO_3_, 0.26 g/L NaH_2_PO_4_, 1.0 g/L glucose, and 2.1 g/L sodium gluconate in nanopure water.^44,45^

### Animals

Female C57BL/6J mice were acquired from The Jackson Laboratory (Bar Harbor, ME). All procedures with mice were reviewed and approved by the Institutional Animal Care and Use Committee at the University of Pittsburgh.

### Device Fabrication

The 2.6 × 2.6 mm^2^ sensor chip, consisting of eight gold interdigitated electrodes (IDEs) as sensing devices, was fabricated on a Si/SiO_2_ substrate. A standard photolithography process was used to pattern the IDEs. A Ti adhesion layer (5 nm) and a Au layer (60 nm) were deposited by e-beam evaporation to form the source and drain contacts. The total thickness of the IDEs and channel length were ∼65 nm and 6 μm, respectively. The sensor chip was then fixed on a standard 40-pin ceramic dual-inline package (CerDIP) using silver paint. Then, the patterned sensing devices were wire-bonded into the package. To secure the bonded gold wires during the sensing experiment, they were covered by poly­(dimethylsiloxane) (PDMS), and the sensor package was heated at 200 °C for 1 h. Deposition of SWCNTs on gold IDEs was performed through AC dielectrophoresis (DEP) using 6 μL of commercial SWCNTs (IsoSol-S100, NanoIntegris) with a concentration of 0.02 mg/mL in toluene by applying an AC frequency of 100 kHz and a bias voltage of 10 V for 3 min. The SWCNT sensor chip was annealed at 200 °C for 12 h. The sensor chip was rinsed with isopropyl alcohol to remove excess SWCNTs. To prepare the antigen-functionalized SWCNT FET biosensors, the carboxyl groups on SWCNTs were activated through EDC/sulfo-NHS coupling by incubating 100 μL of EDC/sulfo-NHS solution at 50 mM/50 mM in PBS on the sensor chip for 30 min. Then, the sensor chip was rinsed with nanopure water to remove the excess EDC/sulfo-NHS solution. Next, 10 μL of antigen solution (1 μg/mL in PBS) was incubated on the sensor chip for 18 h at 4 °C. To prevent the nonspecific interaction of the analyte with SWCNTs, 10 μL of blocking buffer (0.1% Tween 20 and 4% polyethylene glycol in PBS) was incubated on the sensor chip for 30 min. After the sensor chip was washed with nanopure water, it was ready for antibody detection.

### FET Measurements

A liquid-gated FET device configuration was employed to perform FET measurements by a Keithley Source Meter Unit 2400 using a 1 M Ag/AgCl reference electrode as the gate electrode. In FET measurements, source-drain current (*I*_sd_) was collected while the gate voltage (*V*_g_) was sweeping from +0.6 V to −0.6 V with a constant source-drain voltage of 50 mV. A series of antibody solutions from 100 ag/mL to 100 ng/mL for anti-HA and from 100 ag/mL to 10 μg/mL for anti-S and anti-N antibodies were prepared. For calibration measurements in PBS and artificial ISF, the solutions were prepared in PBS and artificial ISF, respectively. The antibody solutions were analyzed from the lowest to the highest concentration.

During the FET measurements, FET transfer characteristics were recorded for each sensing device first after incubation of the sensor chip with 100 μL of blank solution and then after incubation with 100 μL of antibody solution for 10 min. All FET measurements were recorded in nanopure water during anti-SARS-CoV-2 S antibody detection and in the incubated analyte solution during anti-HA antibody detection. To test the response of our devices to the target antibody during the control (using nonfunctionalized devices) and sensing (using antigen-functionalized devices) experiments, the relative response of each sensing device was calculated as *R* = (*I*_sd_ – *I*_0_)/*I*_0_ at *V*_g_ = −0.5 V, where *I*_0_ and *I*_sd_ are the source-drain current before and after analyte incubation, respectively, at an applied gate voltage of −0.5 V.

To investigate the effect of the solvent on the biosensor response, 100 μL of sample media (PBS or artificial ISF) was incubated on the sensor chip for 10 min. The FET transfer characteristics of the devices in the solvent blank were recorded as the baseline. Since 4 orders of magnitude of anti-HA antibody solutions were studied, the blank incubation step was repeated four times. The transfer characteristics were recorded after each solvent blank incubation, and the relative response was calculated.

### Scanning Electron Microscopy Imaging

To characterize the shape of SWCNTs before and after antigen functionalization, scanning electron microscopy (SEM) was used with an accelerating voltage of 1 kV on a ZEISS Sigma 500 VP. SEM samples were coated with a thin layer of PdAu alloy (∼3 nm) by using a Denton Sputter Coater.

### Atomic Force Microscopy

To study height changes, a Bruker Multimode 8 AFM system with a Veeco Nanoscope IIIa controller in the tapping mode was used to take atomic force microscopy (AFM) images of the SWCNT sensor chips before and after functionalization. Gwyddion software was used to process the AFM images and obtain height profiles.

### Raman Spectroscopy

Raman spectra were recorded with the XplorA Raman-AFM/TERS system using a 638 nm (24 mW) excitation laser operating at 1% power. For each sample, the average Raman spectra at 25 locations on the sensor chip was used.

## Results and Discussion

### Characterization of HA-SWCNT FET Devices

The sensor chips were fixed on a standard 40-pin ceramic dual in-line package (40-pin CerDIP) using silver paint and wire-bonded with gold wires, and wires were covered by PDMS (Figure S1). The sensor chip with dimensions of 2.6 × 2.6 mm^2^ consists of the eight patterned gold IDEs with a channel length of 6 μm (Figure 1a). Figure S2 shows the actual image of the sensor chip under an optical microscope and a SEM image of one of the eight patterned IDEs on the sensor chip. The sensors were tested in a liquid-gated FET configuration (Figure 1b). To create a semiconducting transistor channel and a platform for antigen functionalization, SWCNTs were deposited between gold IDEs via DEP of IsoSol-S100 solution. The narrow, well-resolved peaks and low background of the UV–vis–NIR spectrum of IsoSol-S100 solution indicate minimal bundling and removal of metallic CNT species which were consistent with previous studies (Figure S3).^46^ Following DEP and annealing, *I*_sd_–*V*_sd_ curves at different gate voltages were recorded showing the semiconducting behavior of SWCNTs (Figure S4). The FET devices exhibiting desirable FET transfer characteristics showed a maximum current exceeding 10 μA with an *I*_on_/*I*_off_ ratio above 10, where *I*_on_ was taken as *I*_sd_ at −0.6 V_g_ and *I*_off_ as *I*_sd_ at +0.6 V_g_ under a drain bias voltage of 0.05 V (Figure S5a). In contrast, devices with a maximum current below 10 μA and an *I*_on_/*I*_off_ ratio below 10 were classified as nonideal. Therefore, these thresholds were established as the selection criteria for identifying FET devices suitable for subsequent experiments (Figure S5b). The prepared SWCNT FET devices were then functionalized with antigens through an EDC/sulfo-NHS coupling approach. This approach creates covalent chemical bonds between the free amine groups on the antigen and the carboxylic acid groups on the SWCNT sidewalls through EDC/sulfo-NHS coupling. The antigen-functionalized SWCNT FET sensor chip was then exposed to the blocking buffer to prevent nonspecific interactions. The FET transfer characteristics, i.e., source-drain current (*I*_sd_) versus applied gate voltage (*V*_g_), of the HA-SWCNT FET sensors were collected after each fabrication step (Figure 1c). A shift in threshold voltage toward negative gate voltages and a decrease in conductance of FET devices were consistent with the successful HA functionalization.^47,48^ The interaction between the amine groups of the protein and SWCNTs through EDC/sulfo-NHS coupling leads to the donation of electrons to SWCNTs and n-doping.^49^ Blocking the HA-SWCNT FET sensor chip resulted in a further shift of the threshold voltage and decrease in conductance of FET devices. The same behavior in the FET transfer characteristics of S-SWCNT and N-SWCNT FET devices was observed during antigen functionalization and blocking (Figure S6). Comparing the source-drain current with the gate leakage current showed that good insulation between the gate and source-drain electrodes was established as the source-drain current was significantly higher than the gate leakage current (Figure S7).

**Figure 1:**
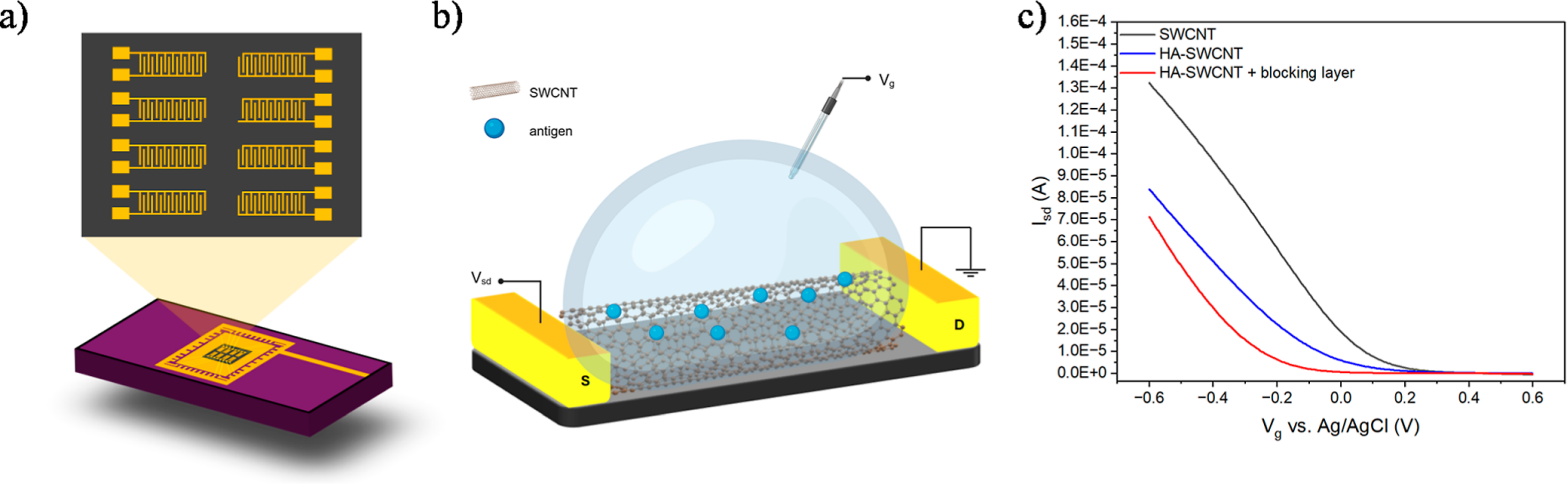
(a) Top: magnification of the sensor chip with eight gold interdigitated devices. Bottom: the wire-bonded sensor chip on a package for field-effect transistor (FET) measurements. (b) Schematic illustration of a liquid-gated antigen-functionalized single-walled carbon nanotube FET biosensor for detection of antibodies. Yellow blocks are interdigitated gold electrodes as the source (S) and drain (D). Gate voltage (*V*_g_) is applied through an Ag/AgCl reference electrode. (c) FET transfer characteristics of a SWCNT FET device before and after antigen (e.g., HA) functionalization and after blocking with 0.1% Tween-20 and 4% polyethylene glycol.

The hemagglutinin decoration on the surface of SWCNTs was characterized by scanning electron microscopy (SEM), Raman spectroscopy, and atomic force microscopy (AFM). Figure 2a shows a SEM image of a SWCNT FET device. The deposition of SWCNTs by DEP between gold IDEs formed a dense interconnected network of carbon nanotubes. As shown in Figure 2b, functionalization of SWCNTs with HA increased the thickness of the SWCNT strands, and some HA aggregation was also observed. Raman spectra of SWCNT and HA-SWCNT FET sensor chips further confirmed the deposition of SWCNTs and the HA functionalization of the sensor chip. The radial breathing mode (RBM) peak corresponds to the uniform diameter distribution of SWCNTs (Figure 2c). The intensity of the RBM band decreased after HA functionalization, showing a disruption in the symmetric structure of SWCNTs due to successful coupling and functionalization. Also, the intensities of the D (1300 cm^–1^) and G (1580 cm^–1^) peaks in the Raman spectra were investigated (Figure 2d). The peak intensity ratio of the D and G peaks (*I*_D_/*I*_G_) increased after HA functionalization, indicating the presence of more defects due to HA functionalization. All spectra were normalized to the Si peak at 507 cm^–1^ (Figure S8). The AFM images of HA-SWCNT (Figure 2e) and SWCNT (Figure 2f) FET devices were collected to investigate the height changes. As shown by the height profiles in Figure S9, the average height of SWCNTs before and after antigen functionalization was 10.46 ± 2.08 and 18.81 ± 5.32 nm, respectively. Therefore, the immobilization of HA antigens on SWCNTs resulted in an average 8.34 ± 5.72 nm increase in height profile after HA functionalization of SWCNTs, which is consistent with previous literature.^50^ This varying average height change shows the difference in orientation of immobilized antigens, which can be standing or lying on SWCNTs. The statistical results proved that there was a significant change in the height of CNT networks at 95% confidence after functionalization with antigens.

**Figure 2:**
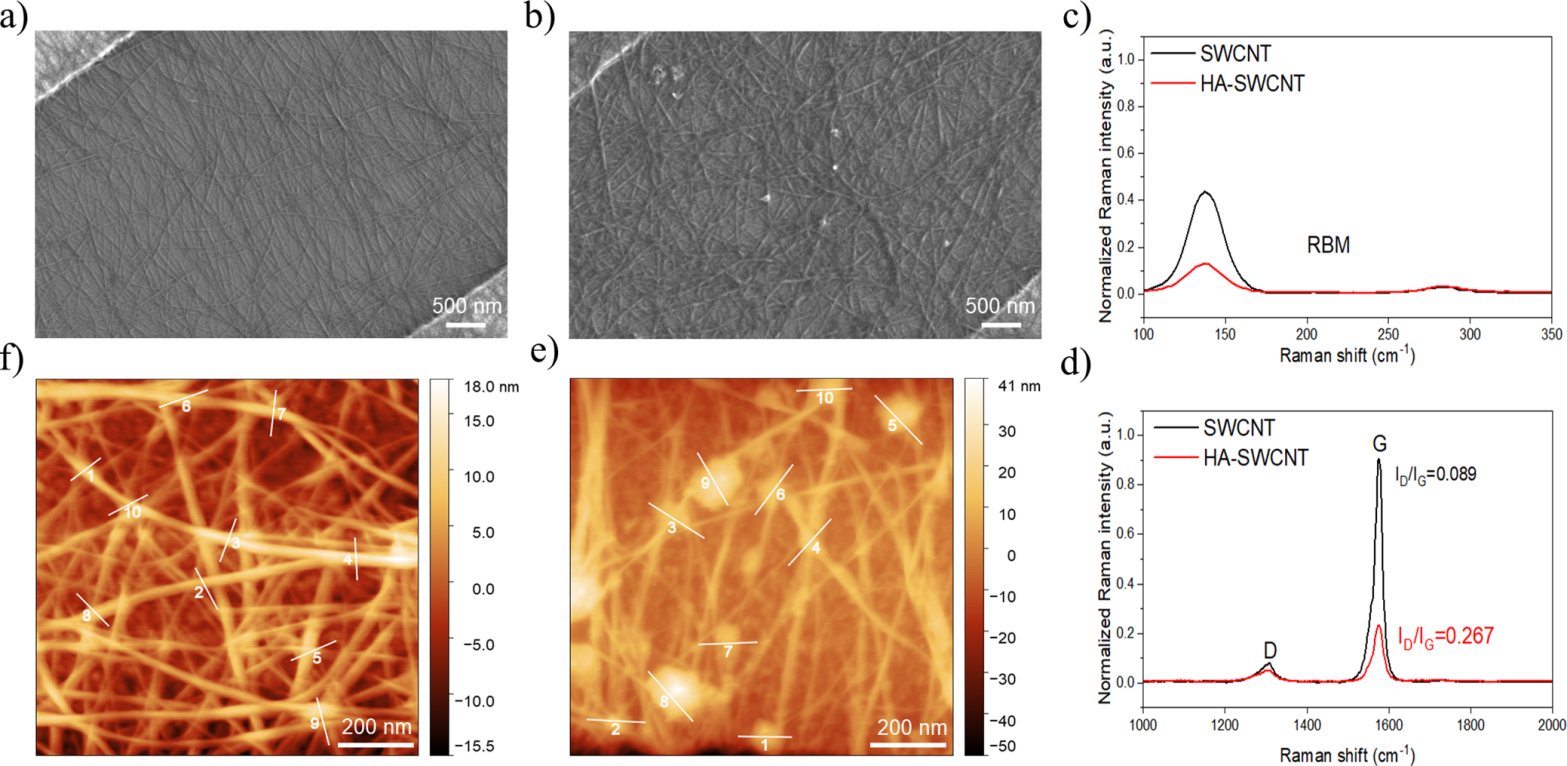
Scanning electron microscopy (SEM) images of (a) SWCNT and (b) HA-SWCNT FET devices. (c) Radial breathing mode (RBM) and (d) D and G peak regions of Raman spectra of the deposited SWCNTs on the sensor chip before and after the immobilization of HA antigens. The Raman spectra were recorded using a 638 nm excitation laser. Atomic force microscopy (AFM) images of (e) HA-SWCNT and (f) SWCNT FET devices.

### Optimization of Device Functionalization

Applying a negative voltage between the gate electrode and the source electrode accumulates cations at the interface between the gate electrode and electrolyte, while anions accumulate at the interface between the CNT channel and the electrolyte. Between these two monolayers, there is a region characterized by exponential decay of the concentration of ions. Each of the two electric double layers behaves as a parallel plate capacitor, screening the rest of the ions from the two interfaces. This phenomenon is called the Debye screening limiting the sensitivity of FET sensors.^51,52^ To increase the sensitivity of the FET biosensor, the amount of antigen as the target receptor on SWCNTs should be optimized to keep the detection sites within the Debye screening length and provide maximum numbers of binding sites for antibody detection.^53^ To do this, 10 μL of antigen solution at concentrations of 0.1, 1, 10, and 100 μg/mL was incubated on the SWCNT FET sensor chip for 18 h at 4 °C. After blocking, the prepared FET sensor chip was exposed to 100 ng/mL antibody solution in PBS, and the relative response was monitored. Figure S10a shows that the FET biosensor response increased when the amount of antigen increased from 0.1 to 1 μg/mL, proving more binding sites for the interaction of receptors and target analyte. However, as the amount exceeded 1 μg/mL, the relative response decreased. These results may be indicative of multilayer formation at higher loadings with recognition sites positioned farther away from the SWCNT surface, where interactions between antigens and antibodies happen beyond the Debye screening length and therefore do not affect the mobility of charge carriers inside nanotubes.

Following the established procedure in our previous publication, all FET measurements for anti-N and anti-S detection were performed in nanopure water to eliminate the effect of the Debye screening.^48^ Although utilizing nanopure water improves sensitivity, it complicates the sensing procedure, requiring several washing steps. Moreover, this approach prevents real-time sensing as the sample solution must be removed from the sensor. In contrast, direct FET measurements in antibody samples may decrease sensitivity but simplify the sensing procedure, facilitating real-time sensing. As different concentrations of the gating media have different ionic strengths and, consequently, different Debye screening lengths, the concentrations of the gating media should be optimized. The FET transfer characteristics of the HA-SWCNT FET biosensor were recorded using different concentrations of PBS (0.01 0.1, and 1× PBS) as the gating electrolyte. The calibration plot in Figure S10b shows that the concentration of PBS does not significantly affect the biosensor response. The reasons for this can be attributed to several factors. The small size of the covalently attached antigens makes it possible to keep the binding sites within the Debye length. Although the overall sizes of receptors and analyte molecules may exceed the Debye length, the location of binding sites on antigens can allow antigen–antibody binding within the Debye length based on the orientation of antigens on the SWCNTs.^54^ In addition, the proposed sensing mechanism based on the electron-donating activity of the receptor located in close proximity to the SWCNT surface is not affected by the Debye length screening. Therefore, the proposed FET biosensor enables direct FET measurements and provides real-time sensing by eliminating the need to remove the sample solution from the sensor and several washing steps (Figure S11).

To optimize the analyte incubation time on the FET biosensor, the HA-SWCNT FET biosensor was incubated with 1 fg/mL anti-HA and FET transfer characteristics were recorded over 15 min. The sensor response increased continuously during the first 10 min and plateaued thereafter (Figure S12a). To confirm this trend across different analyte concentrations, an HA-SWCNT FET device was incubated with a concentration series of anti-HA for 12 min. The results consistently indicated that an incubation time of 10 min was sufficient to achieve a stable sensor response (Figure S12b).

The sensor is designed to operate in biological samples within the pH range of 6.5–7.4. Previous studies have shown that pH variations within this narrow range have a negligible effect on sensor performance, as they are much smaller than the response observed even at the lowest analyte concentration.^55,56^ Therefore, the observed sensor response is not significantly affected by the pH changes. Accordingly, all PBS and artificial ISF solutions used in this work were buffered to pH 7.4.

### Detection of Anti-SARS-CoV-2 Antibodies

To demonstrate the broad applicability of our SWCNT FET biosensors, we rapidly developed new biosensors to detect anti-SARS-CoV-2 nucleocapsid (anti-N) and anti-SARS-CoV-2 spike (anti-S) antibodies. Functionalization of SWCNTs with SARS-CoV-2 nucleocapsid (N) and SARS-CoV-2 spike (S) proteins provided two different FET biosensors. The N-protein-functionalized SWCNT (N-SWCNT) and S-protein-functionalized SWCNT (S-SWCNT) FET biosensors were applied for the analysis of anti-N and anti-S antibodies, respectively. Figure 3a shows FET transfer characteristic curves (*I*–*V*_g_) of an N-SWCNT FET device upon exposure to different concentrations of anti-N antibody from the lowest to the highest concentration. The prepared N-SWCNT FET biosensor was able to detect the analyte with high sensitivity in a linear concentration range of 100 ag/mL to 100 ng/mL in PBS (Figure 3b). As shown in Figure 3c, S-SWCNT FET transfer characteristic curves display direct trends in correlation with different concentrations of anti-S antibody from the lowest to highest concentration. The prepared S-SWCNT FET biosensor sensitively detected the analyte in a linear concentration range of 100 ag/mL to 1 ng/mL (Figure 3d). The conductance increased with an increasing concentration of antibody. This can be attributed to the charge redistribution upon antigen–antibody interactions leading to a reduction in the overall electron donation of proteins, thus p-doping SWCNTs.^52,57,58^ The increase in the conductance of FET devices resulted in higher relative responses. To eliminate the Debye screening effect, nanopure water was utilized as the gating electrolyte for the FET measurements. Although a good sensing performance was achieved, this approach required a separate washing step to add the gating electrolyte, which complicated the sensing process, and as a result, this approach was not suitable for real-time sensing.

**Figure 3:**
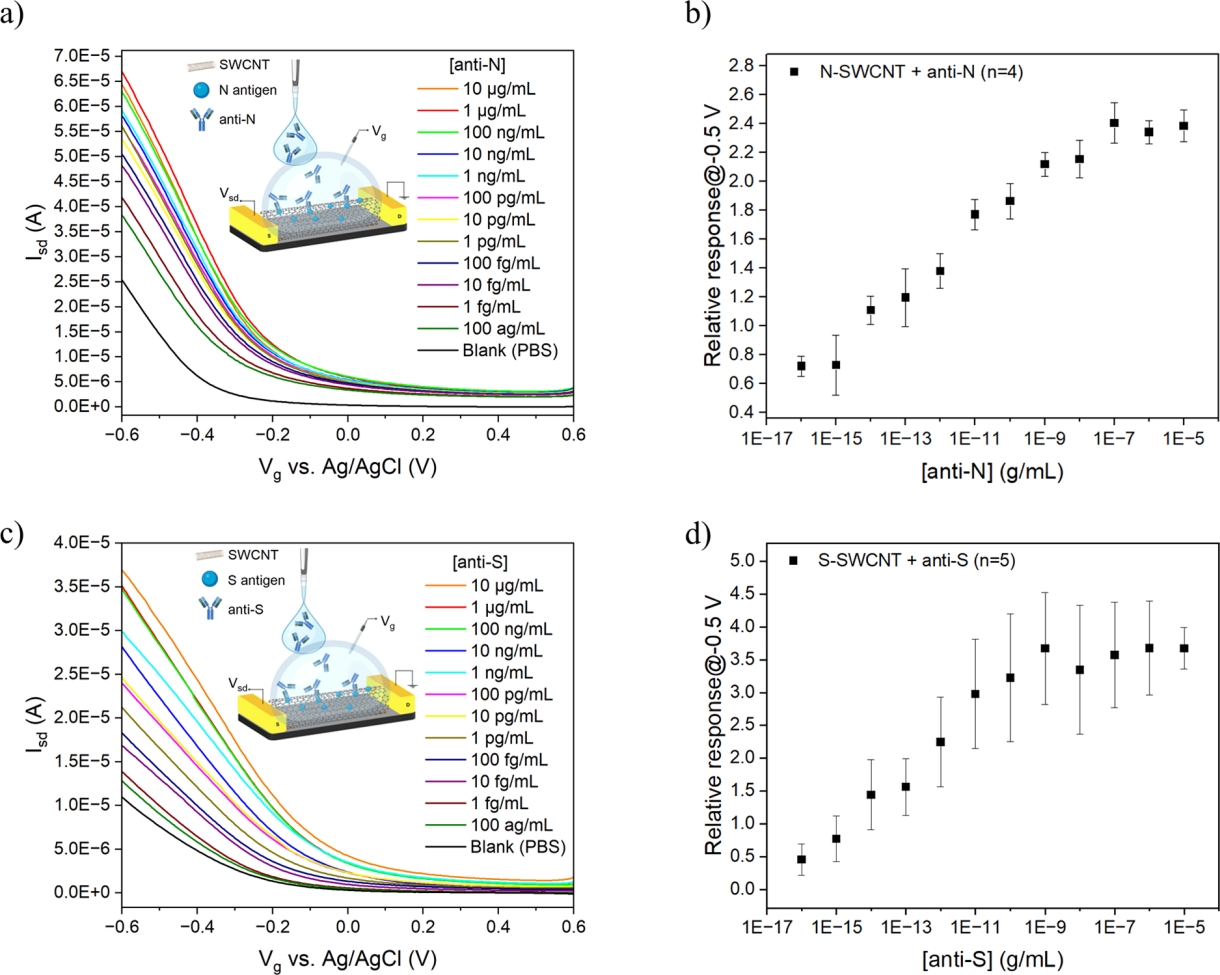
Detection of anti-SARS-CoV-2 Nucleocapsid (anti-N) and anti-SARS-CoV-2 Spike (anti-S). (a) FET characteristic curves of a SARS-CoV-2 N protein-functionalized SWCNT (N-SWCNT) FET device upon exposure to an increasing concentration of anti-N antibody. (b) Calibration plot for anti-N antibody detection. (c) FET characteristic curves of a SARS-CoV-2 S protein-functionalized SWCNT (S-SWCNT) FET device upon exposure to an increasing concentration of anti-S antibody. (d) Calibration plot for anti-S detection. All data points plotted in the calibration plots are mean ± standard error. The number of devices (*n*) used for sensing is indicated in the parentheses in the legend.

### Detection of Anti-HA Antibodies in PBS

The optimizations of the sensor configuration allowed the sensors to overcome the Debye screening effect and detect antibodies directly in the samples by the immobilization of small antigens as receptors on the SWCNTs. By neutralizing the Debye screening effect, FET measurements were carried out in the sample solution. This approach eliminated the need for washing steps to remove the sample and add the gating electrolyte. As a result, this simplified the sensing procedure and provided a platform for real-time sensing. To investigate the performance of the FET biosensor in PBS media, anti-HA antibody calibration samples were prepared in PBS. As shown in Figure 4a, the FET transfer characteristic curves (*I*–*V*_g_) displayed direct trends upon exposure to anti-HA antibody calibration samples from the lowest to the highest concentration. The calibration plot for anti-HA antibody detection by the HA-SWCNT FET biosensor shows that the designed biosensor responded proportionally to the logarithm of the concentration over a desirable linear range from 100 ag/mL to 100 ng/mL (Figure 4b). Using an SWCNT FET biosensor (without HA attachment), a control experiment was conducted to investigate the biosensor specificity. In the absence of HA receptors on SWCNTs, the recorded responses toward different concentrations of anti-HA antibody were significantly different (Figure 4b). The effect of solvent on the biosensor response was studied by four times incubation of PBS on the HA-SWCNT FET biosensor. As shown in Figure 4b, PBS caused no significant conductance change, indicating that the biosensor response results from the interactions between HA and the anti-HA antibody.

**Figure 4:**
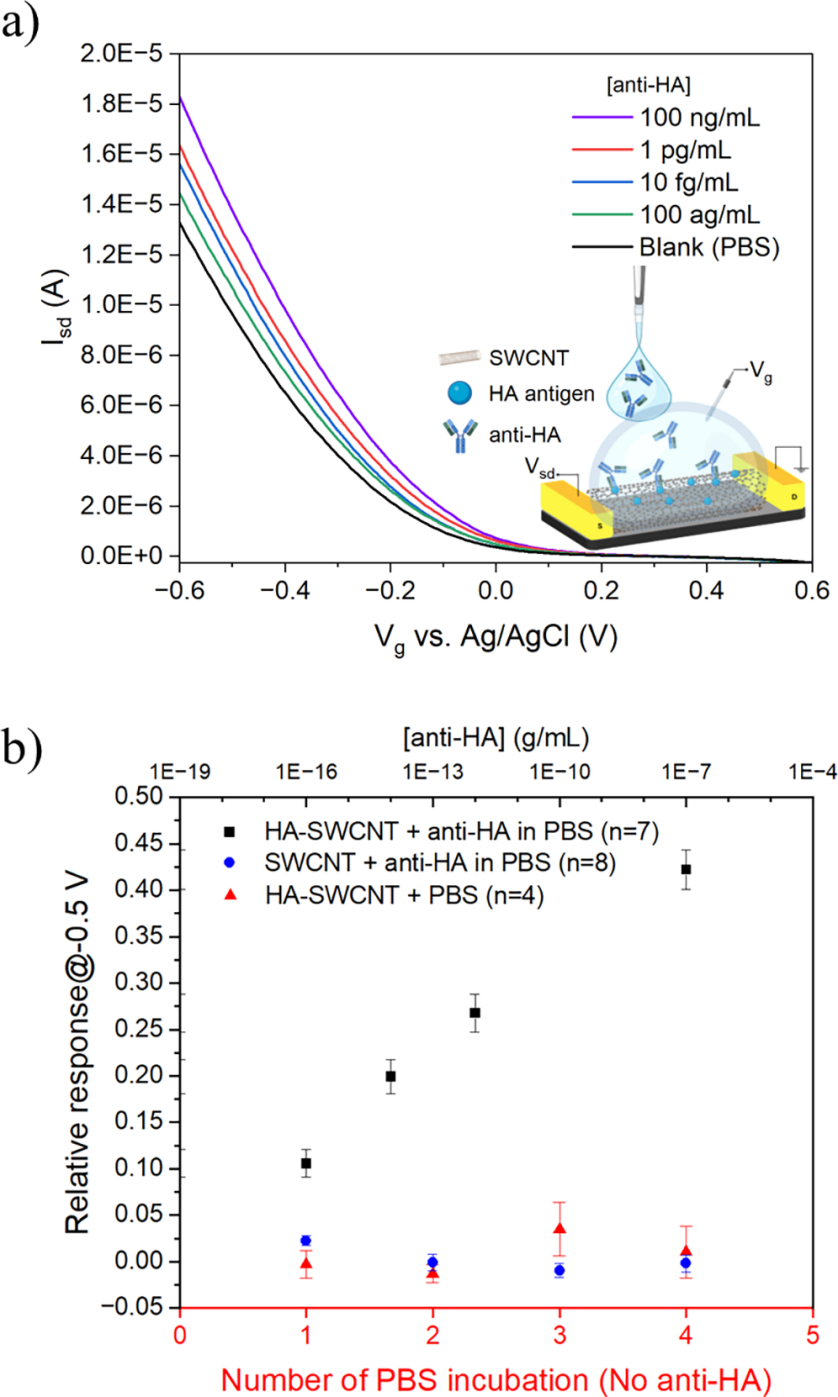
Detection of anti-hemagglutinin (anti-HA) antibody in PBS. (a) FET characteristic curves of a HA-SWCNT FET device upon exposure to an increasing concentration of anti-HA antibody in PBS. (b) Calibration plot for anti-HA detection in PBS (black), testing different concentrations of anti-HA on the nonfunctionalized SWCNT FET sensor chip as a control experiment (blue) and the effect of multiple incubation of the blank on the FET sensor (red). All data points plotted in the calibration plots are mean ± standard error. The number of devices (*n*) used for sensing is indicated in the parentheses in the legend.

### Detection of Anti-HA Antibodies in Artificial Interstitial Fluid (ISF)

To investigate the capability of the FET biosensor in detecting target antibodies in ISF media, anti-HA antibody calibration samples were prepared in an ISF. Figure 5a demonstrates that FET transfer characteristic curves (*I*–*V*_g_) during detection of anti-HA antibody in artificial ISF exhibited similar behavior to that observed in PBS. The prepared HA-SWCNT FET biosensor was able to detect the analyte in a dynamic linear range of 100 ag/mL to 100 ng/mL (Figure 5b). The specificity of the biosensor for the detection of anti-HA antibody in artificial ISF and the effect of artificial ISF on the biosensor response were monitored in the same way as those for PBS media. The recorded responses toward different concentrations of anti-HA antibody in the absence of HA antigens were negligible, and the HA-SWCNT FET biosensor did not show any significant relative response upon subsequent incubation with artificial ISF (Figure 5b). These statistical results revealed the successful performance of the biosensor in detecting the anti-HA antibody in a complex environment.

**Figure 5:**
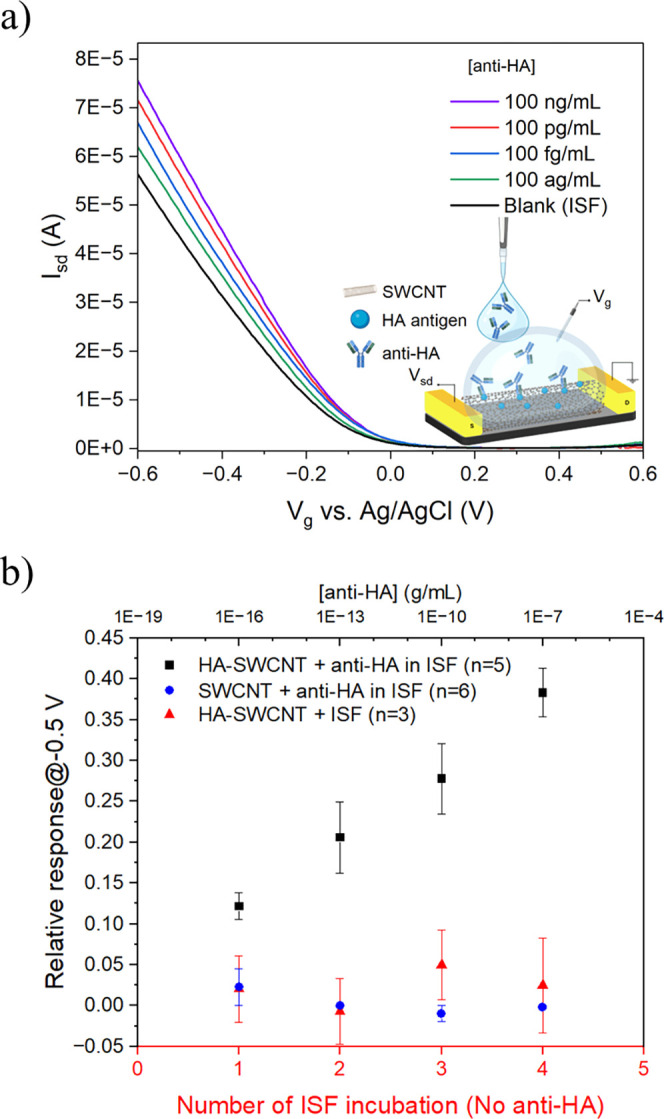
Detection of anti-hemagglutinin (anti-HA) antibody in artificial interstitial fluid (ISF). (a) FET characteristic curves of a HA-SWCNT FET device upon exposure to an increasing concentration of anti-HA antibody in artificial ISF. (b) Calibration plot for anti-HA detection in artificial ISF (black), testing different concentrations of anti-HA on the nonfunctionalized SWCNT FET sensor chip as a control experiment (blue) and the effect of multiple incubation of the blank on the FET sensor (red). All data points plotted in the calibration plots are mean ± standard error. The number of devices (*n*) used for sensing is indicated in the parentheses in the legend.

### Limit of Detection of the Designed Biosensors

The limit of detection (LOD) was calculated following the International Union of Pure and Applied Chemistry (IUPAC) definition.^59^ First, the smallest sensor response that could be reliably distinguished (*x*_L_) was calculated using the equation $x_{L}={\mathrm{x̅}}_{B}+ks_{B}$ . In this equation, ${\mathrm{x̅}}_{B}$ represents the mean of blank measurements, *s*_B_ is the standard deviation of the blank measurements, and k is set to 3 to achieve a confidence level of 99.6%. For the S-SWCNT and N-SWCNT biosensors, the lowest concentration tested was treated as the blank, and the corresponding *x*_L_ values were determined based on the smallest sensor response at that concentration. By interpolating the *x*_L_ values on the fitted calibration curves, the corresponding LODs were determined to be 1.41 and 4.82 fg/mL for the detection of anti-N and anti-S antibodies in 1× PBS, respectively (Figure S13).

Lower detection limits can also be calculated by comparing the analytical signal to the statistical fluctuations of the blank signal. For example, when the S-SWCNT biosensors were incubated with PBS alone as the solvent blank, a lower *x*_L_ value was obtained. By interpolating this *x*_L_ value on the fitted calibration curve, an LOD of 20.6 ag/mL was determined for the detection of the anti-S antibody (Figure S14).

For HA-SWCNT sensors, the HA-SWCNT FET sensor chips were first incubated with blank solutions and FET characteristic curves were recorded 20 times (Figure S15). By interpolating *x*_L_ values on the fitted calibration curves, the corresponding LODs were determined to be 0.20 and 7.0 ag/mL for the detection of anti-HA antibody in 1× PBS and artificial ISF, respectively (Figure S16).

Each FET sensor chip contains up to eight working devices, and the calculated LOD values are ultimately limited by device-to-device variations. By selecting the best-performing FET device on the sensor chip, we were able to achieve lower LODs. For single devices, interpolation of the *x*_L_ values on the fitted calibration curves yielded LODs of 0.096 and 0.32 ag/mL for the detection of anti-HA in 1× PBS and artificial ISF, respectively (Figure S17).

As part of the calibration process, the HA-SWCNT FET sensor chip was incubated with multiple analyte solutions to generate the calibration curve. The LOD of the overall method was determined by repeatedly incubating the FET sensor with the blank solution. The absolute mean of the sensor responses to the blank and the standard deviation of these responses were calculated. Using this approach, interpolation of the *x*_L_ values on the fitted calibration curves yielded LODs of 2.0 and 8.0 ag/mL for the detection method in 1× PBS and artificial ISF, respectively (Figure S18).

### Selectivity and Stability of the Designed Biosensors

For selectivity experiments to avoid any cross-reactivity in our experiments, we intentionally chose orthogonal protein antigens (i.e., two antigens without any common epitopes, so no antibodies could recognize both proteins). To investigate the selectivity of the N-SWCNT FET biosensor toward anti-N antibodies, a sensor chip was used to analyze anti-N and anti-S solutions. The designed N-SWCNT showed significantly higher responses to anti-N antibody in comparison to anti-S antibody (Figure 6a). The selectivity of the FET biosensor was examined by monitoring the HA-SWCNT biosensor response after exposure to different proteins with the same concentration range as anti-HA. The hydrophobic nature of SWCNTs can promote nonspecific adsorption of hydrophobic proteins, potentially affecting the selective performance of the biosensor. In our study, this effect was minimized using a blocking step and careful surface functionalization, which reduced nonspecific binding and enhanced the recognition layer specificity. These strategies helped mitigate the influence of protein hydrophobic domains on SWCNTs and limited nonselective interactions between interfering proteins and the receptors. The FET biosensor showed remarkably different responses to anti-HA compared with other proteins, confirming that the biosensor response to the analyte is attributed to the specific interactions between antigen and its antibody (Figure 6b). This specific interaction provides a selective sensor for antibody detection.

**Figure 6:**
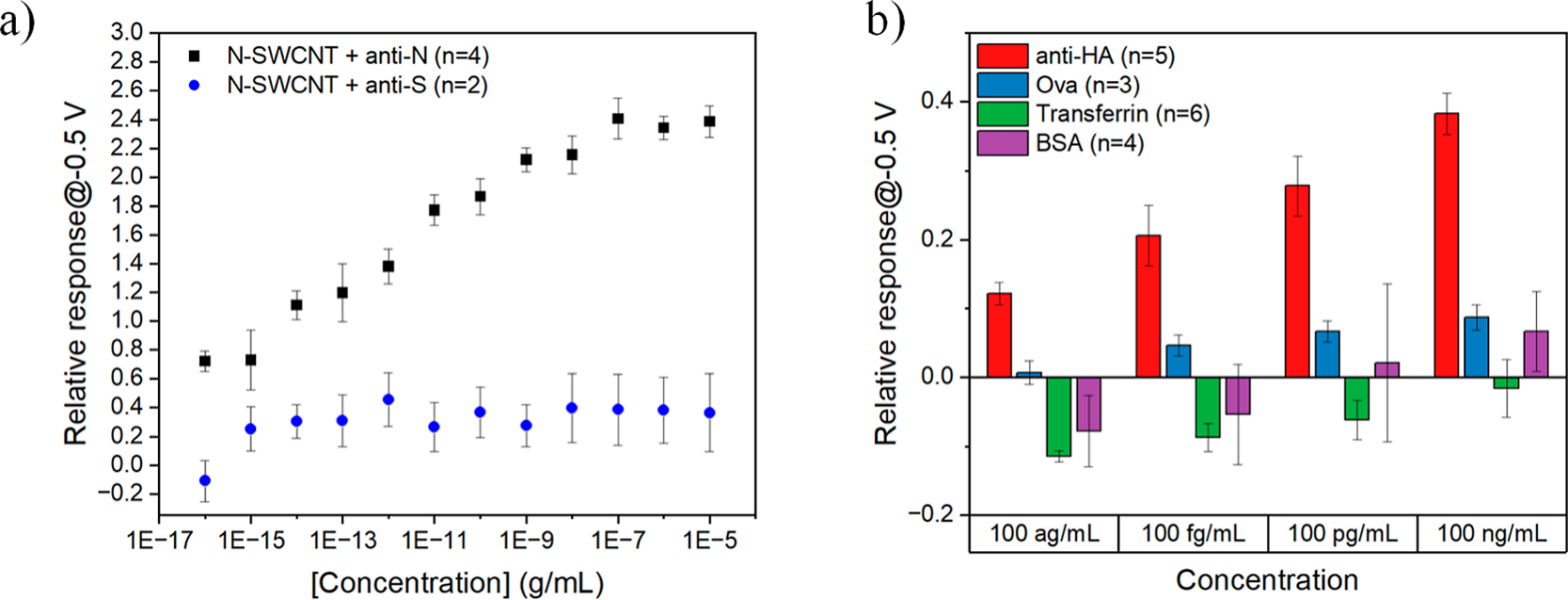
Selectivity tests of FET biosensors. (a) Relative response of the N-SWCNT FET biosensor to different concentrations of anti-N and anti-S antibodies. (b) Relative response of the HA-SWCNT FET biosensor to different concentrations of anti-HA antibody, ova, transferrin, and BSA (all proteins are in artificial ISF). All plotted relative responses are mean ± standard error. The number of devices (*n*) used for sensing is indicated in the parentheses in the legend.

To evaluate the storage stability of the prepared sensor chips, HA-SWCNT FET biosensors were stored under four different conditions for 8 days. During this period, the FET transfer characteristics were periodically recorded to monitor the source-drain current. The biosensors were first placed under ambient conditions for 30 min to ensure that the FET devices are at room temperature for FET measurements. After storage, HA-SWCNT FET biosensors were tested against a concentration range of the anti-HA antibody. While the source-drain current did not change significantly in any of the stored FET biosensors, only HA-SWCNT FET devices stored at −20 °C retained reliable sensing performance. These results indicate that the prepared FET sensors can retain their analyte detection capability after prolonged storage. Moreover, these findings suggest that antigen-functionalized FET devices may undergo receptor deactivation during storage, even if their FET transfer characteristics remain largely unchanged (Figure S19).

### Performance Evaluation of SWCNT FET Biosensors

While most diagnostic methods focus on detecting pathogens to identify diseases, this work introduces a platform designed to detect antibodies generated after viral infection or vaccination. This method enables the identification of infected individuals soon after exposure to a virus or convenient and accurate monitoring of vaccine-induced antibodies. Our FET biosensor demonstrates a promising limit of detection, wide linear concentration range, and rapid response time compared to previously reported techniques (Table S1) and FET-based sensors (Table S2). The sensor performance metrics were significantly improved by applying a real-time sensing approach.

### Direct Validation of SWCNT FET Biosensors with Biological Samples

With the ultimate goal of developing wearable platforms for antibody detection via skin, the rapid antibody detection performance of our SWCNT FET biosensors was evaluated with homogenized skin samples from mice immunized with a SARS-CoV-2 spike vaccine. The S-SWCNT FET biosensor was exposed to two different homogenized skin tissue lysate samples collected from immunized mice. First, the S-SWCNT was incubated with diluted naive mouse samples as blank, and FET was recorded. Then, the diluted immunized mouse samples were incubated on the S-SWCNT FET biosensor and FET characteristic curves were collected to calculate relative response of the biosensor toward antibody in mouse skin (Figure 7). To enable on-site detection of anti-S antibody, the FET transfer characteristics were recorded by utilizing a portable dual-channel potentiostat. Comparison of the FET characteristic curves of a SWCNT FET device suggests that no significant difference is observable between the portable potentiostat and laboratory high-precision sourcemeters (Figure S20). The developed SWCNT FET biosensor holds strong potential for rapid and point-of-care screening, particularly for monitoring antibody responses after vaccination or infection. Its high sensitivity, label-free operation, and potential for miniaturization make it an attractive candidate for clinical diagnostics. However, several challenges must be addressed before a successful translation. Matrix effects in complex biological fluids (e.g., serum, saliva, or whole blood) may reduce sensitivity because of nonspecific adsorption and high ionic strength. Rigorous regulatory and clinical validation is required to ensure reproducibility, reliability, and safety before deployment in healthcare settings. Finally, practical use will depend on the integration with user-friendly platforms, including portable and automated devices, to enable high-throughput or point-of-care testing.

**Figure 7:**
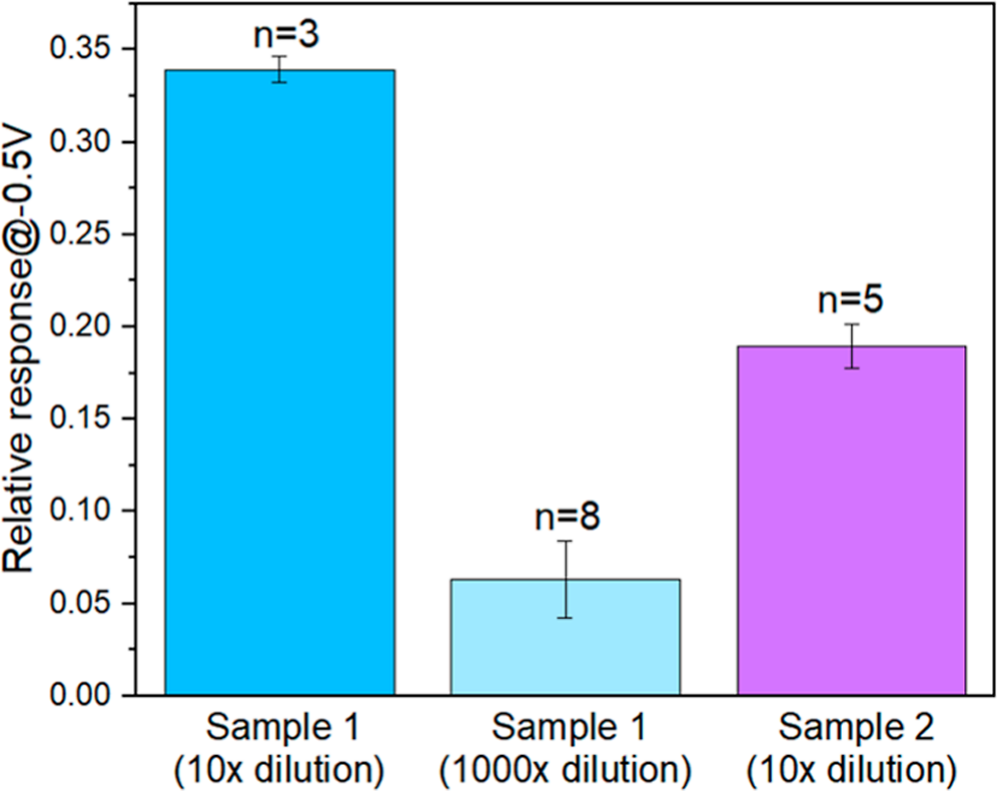
Analysis of skin tissue lysate samples collected from immunized mice with antibodies against SARS-CoV-2 spike proteins. Relative responses of the S-SWCNT FET biosensor to two different skin tissue lysate samples after 10- and 1000-times dilution were recorded. All plotted relative responses are mean ± standard error. The number of devices (*n*) used for sensing is indicated.

## Conclusion

In this study, FET biosensors based on semiconducting SWCNTs functionalized with target protein antigens (e.g., HA antigen of H1N1 virus and spike protein or nucleocapsid protein of SARS-CoV-2 virus) for specific detection of the corresponding anti-HA, anti-S and anti-N antibodies were fabricated. The N-SWCNT and S-SWCNT FET biosensors demonstrated a large and selective response to the corresponding antibody, with implications that the HA-SWCNT FET biosensor has a better LOD value that is at the ag/mL level. The designed FET biosensor was exposed to antibodies in different matrices, and it showed reliable performance in the detection of the analyte in artificial ISF and ex vivo samples. The performance of FET-based sensors can be hindered by the Debye length effect, particularly when larger biomolecules are detected in high ionic strength media. In addition, achieving consistent CNT deposition remains a challenge, which can limit device reproducibility. The fabrication process often suffers from low yield and batch-to-batch variations, posing difficulties for large-scale production. Finally, despite the use of blocking layers, nonspecific adsorption in complex biological samples may still influence the measurement accuracy. Overall, designing effective biosensors for the detection of antibodies opens the opportunity for real-time monitoring of antibody levels in the body after a vaccine. This detection assay approach has the potential to be applied to the detection of other biological molecules.

## Supplementary Material





## Notes

Photos of sensor preparation on the package, optical microscopy and SEM images of FET devices, UV–vis–NIR spectrum of IsoSol-S100 solution, *I*_sd_–*V*_sd_ curve of the SWCNT FET device, FET characteristic curves during the fabrication process of FET biosensors, comparison of source-drain current with the gate leakage current, Raman spectra before and after antigen functionalization of SWCNTs, height profiles of SWCNT FET devices before and after antigen functionalization, optimization of antigen functionalization of SWCNTs and the concentration of gating electrolyte, schematic illustration of the sensing procedure, incubation time optimization, limit of detection values based on fitting of calibration curves, stability evaluation, and comparison of results of the portable potentiostat and the sourcemeter (PDF)
FET measurement (MP4)
