# Supplementary material for: Monitoring Vaccine-Induced Antibody Levels Using Carbon Nanotube-Based Field-Effect Transistors
Source: Anal Chem. 2025 Nov 3;97(49):27102–12. doi: 10.1021/acs.analchem.5c03817 (PMC12713613; doi:10.1021/acs.analchem.5c03817)
Supplement: Supplementary file 1 [file ac5c03817_si_001.pdf]

Supporting Information

Monitoring Vaccine-Induced Antibody Levels Using Carbon Nanotube-Based Field-Effect Transistors

Amir Amiri[1], Wenting Shao[1], Zidao Zeng[1], Ashish Dhayani[2], Stephen C. Balmert[2], Louis D. Falo Jr[2,3,4,5], Emrullah Korkmaz[2,3], Alexander Star[1,3]*

[1]Department of Chemistry, University of Pittsburgh, Pittsburgh, Pennsylvania 15260, United States

[2]Department of Dermatology, University of Pittsburgh School of Medicine, Pittsburgh, Pennsylvania 15213, United States

[3]Department of Bioengineering, University of Pittsburgh, Pittsburgh, Pennsylvania 15261, United States

[4]Clinical and Translational Science Institute, University of Pittsburgh, Pittsburgh, Pennsylvania 15213, United States

[5]The McGowan Institute for Regenerative Medicine, University of Pittsburgh, Pittsburgh, Pennsylvania 15219, United States

Table of Contents

Figure S1. Sensor preparation……………...………………………………………..………S3

Figure S2. Characterization of a sensor chip and gold interdigitated devices…………………...S3

Figure S3. UV-VIS-NIR spectrum of IsoSol-S100 solution at 0.02 mg/mL in toluene…………S4

Figure S4. The I_{sd}-V_{sd} curve of a SWCNT deposited FET device at different gate voltages..........S5

Figure S5. The selected criteria for suitable FET devices..…………………………………..…S6

Figure S6. FET transfer characteristics of SWCNT FET devices before and after functionalization………………………………………………………………………………S7

Figure S7. The negligible gate leakage current (Ig) of a HA-SWCNT FET device in comparison to source-drain current (I_{sd}). ……………………….………………………………….………...S7

Figure S8. Raman spectra of SWCNTs deposited sensor chips before and after the immobilization of HA antigens……………………………..…………………………………...…………..S8

Figure S9. Extracted height profiles from AFM images. ………………..……………...…….S9

Figure S10. Optimization of HA functionalization and the gate electrolyte. …………...………S10

Figure S11. The FET biosensing procedure…………………………………………………S11

Figure S12. Incubation time optimization. ……………………………………...…………S12

Figure S13. LOD calculations for the detection of anti-SARS-CoV-2 nucleocapsid (anti-N) and anti-SARS-CoV-2 spike (anti-S) antibodies. ……………………………………..……….S13

Figure S14. LOD calculations for the detection of anti-SARS-CoV-2 spike (anti-S) antibody.S14

Figure S15. FET characteristics of multiple measurements after 10 min incubation of the HA-SWCNT FET sensor with (a) 100 µL 1× PBS and (b) 100 µL artificial ISF..............................S15

Figure S16. LOD calculations for the detection of anti-HA in 1× PBS and artificial ISF……....S16

Figure S17. LOD calculations for the detection of anti-HA on a single FET device……………S17

Figure S18. LOD calculations for the applied detection method……………………………….S18

Figure S19. Stability evaluation……………………………………………………...…...S19

Figure S20. Comparing the portable potentiostat and laboratory high precision sourcemeter in recording FET characteristic curves of a SWCNT FET device…………………………......S19

Table S1. Comparing the proposed biosensor with previously reported techniques……………S20

Table S2. Comparing the SWCNT FET biosensor with other FET-based sensors designed for antibody detection…………………………………………………………………………S21

References……………………...……………………………………….………………...…S22

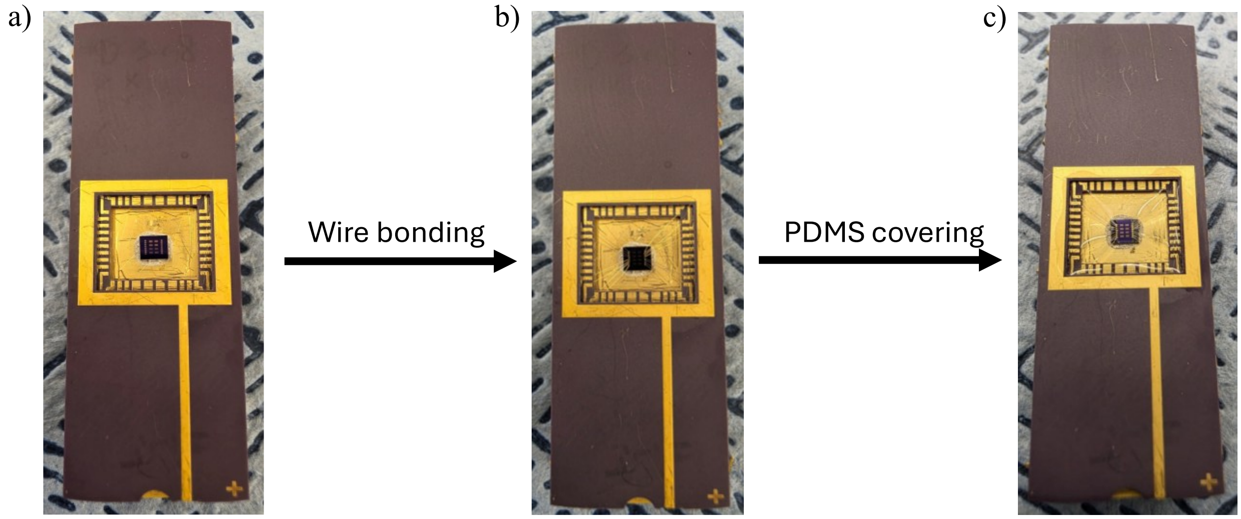

Figure S1. Sensor preparation. (a) The sensor chip fixed on a CerDIP using silver paint. (b) Wire-bonded sensor chip into the package. (c) Bonded gold wires covered by polydimethylsiloxane (PDMS).

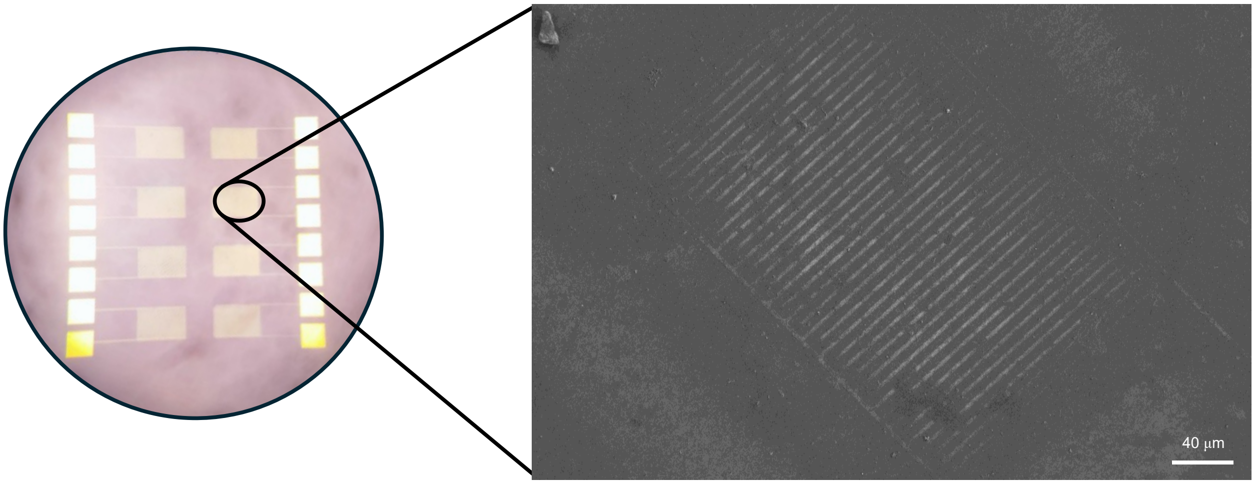

Figure S2. Characterization of a sensor chip and gold interdigitated devices. (a) Optical microscopy image of a sensor chip. (b) SEM image of a SWCNT FET device on the sensor chip.

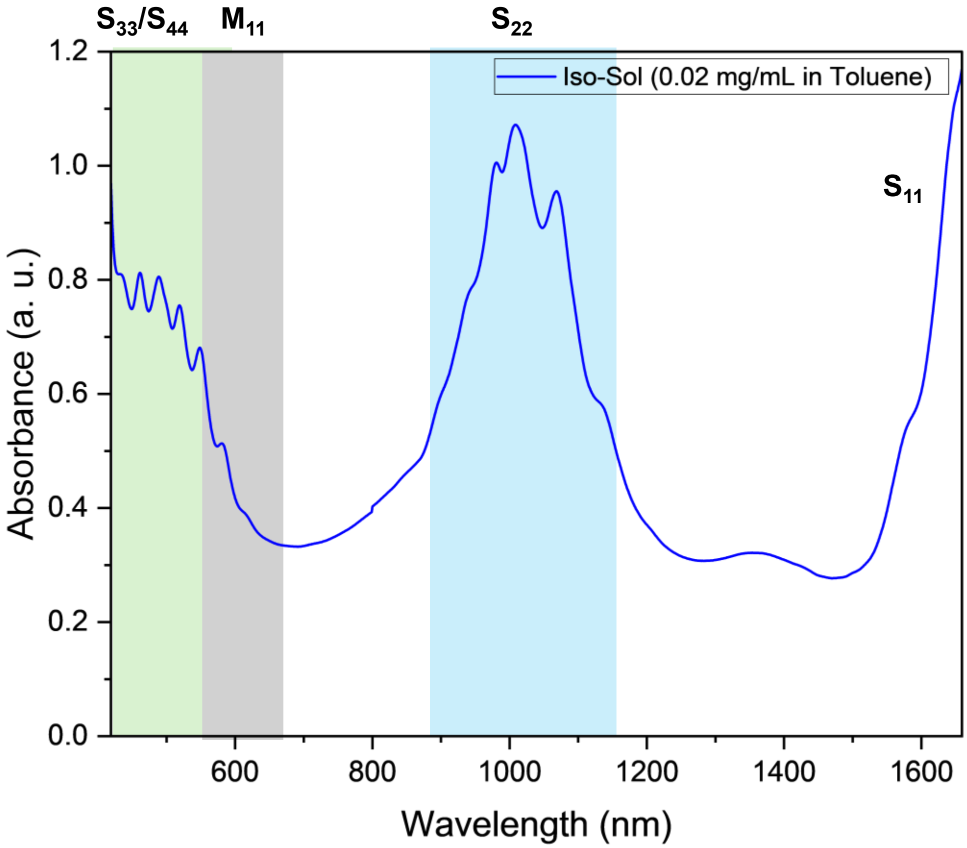

Figure S3. UV-VIS-NIR spectrum of IsoSol-S100 solution at 0.02 mg/mL in toluene.

The UV–Vis–NIR spectra of semiconductor–enriched SWCNTs can be interpreted as arising from discrete excitonic transitions which are characteristic to semiconducting single-walled carbon nanotubes. In the visible region, the weaker features corresponding to higher energy transitions (S_{33}) and, if present, residual metallic M_{11} bands are observed. The most prominent absorption peaks are in the near IR which correspond to S_{22} (~900–1100 nm) for large diameter nanotubes, which include the sharp (10, 9) resonance which dominates the spectrum due to high chiral purity achieved via conjugated polymer wrapping. The corresponding S_{11} transition for the (10,9) is observable around 1550–1600 nm (~ 6800–6530 cm^{-1}). The S_{11} transition is associated with the bandgap of this 1.3 nm-diameter nanotube.[S1]

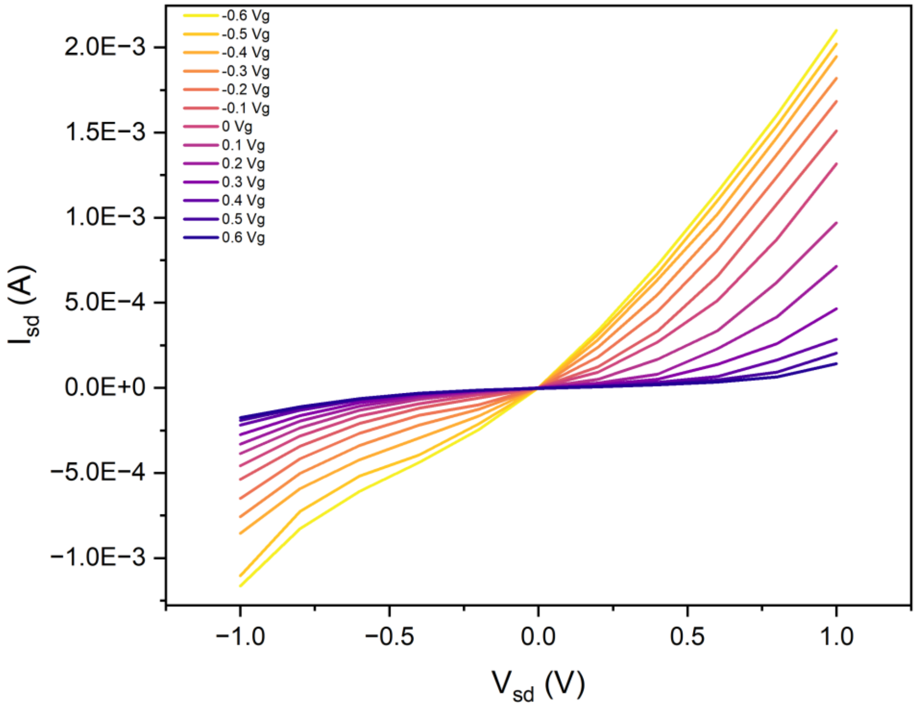

Figure S4. The I_{sd}-V_{sd} curves of a SWCNT deposited FET device at different gate voltages.

When the source–drain bias voltage (V_{sd}) is applied, sweeping the gate voltage (V_g) toward more negative values results in an increase in the source–drain current (I_{sd}). This response is characteristic of p-type semiconducting SWCNTs. A more negative gate potential attracts positive charges toward the reference electrode, leading to an accumulation of negative charges on the SWCNT surface. This process enhances p-type doping, which increases the hole concentration (the major charge carriers) in the SWCNT channel, thereby elevating I_{sd}.

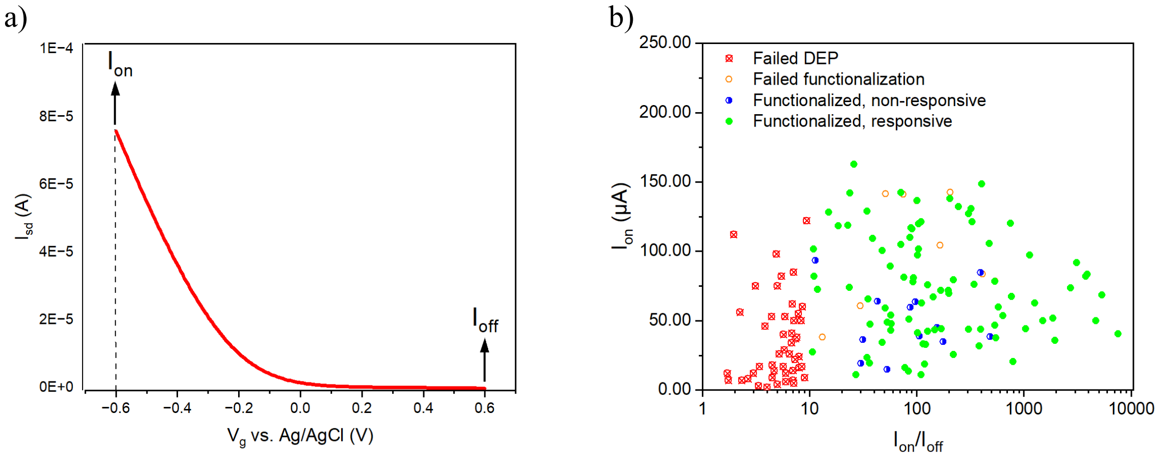

Figure S5. The selected criteria for suitable FET devices. (a) The desired FET transfer characteristics curve. (b) The statistical distribution of fabricated devices in terms of I_{on} and I_{on}/I_{off}.

In this project, 22 packages comprising 176 devices were fabricated using DEP. Among these, 125 devices (71.0%) were successfully deposited with SWCNTs. Of the working devices, 115 (92.0%) were properly functionalized and prepared for sensing experiments, while 10 devices did not meet the defined criteria. Of the functionalized devices, 103 (89.6%) demonstrated reliable performance, whereas 12 did not. The average on-current for the prepared devices at $V_g = -0.6$ V was 80.6 μA, and the average I_{on}/I_{off} ratio was 597.

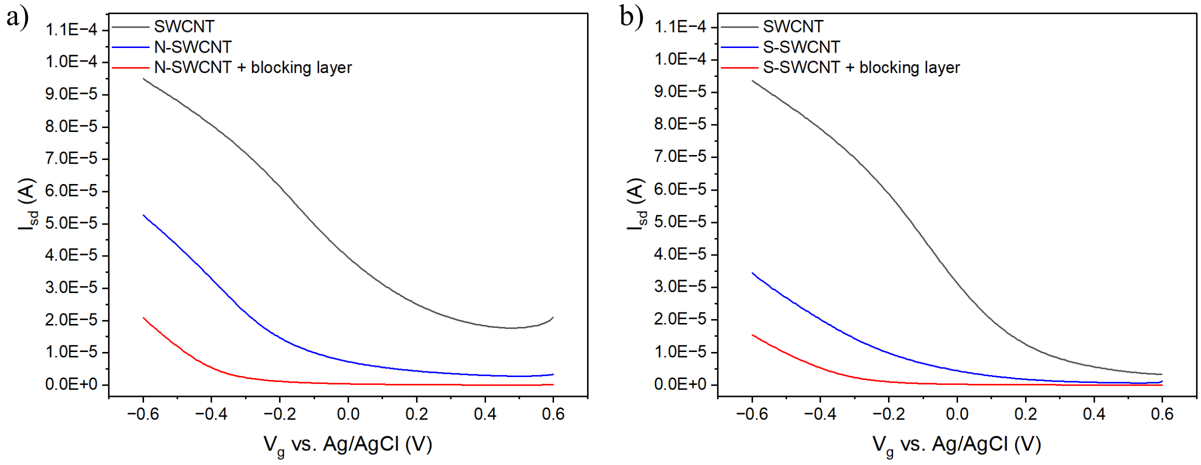

Figure S6. FET transfer characteristics of SWCNT FET devices before and after functionalization of SWCNTs with (a) SARS-CoV-2 nucleocapsid (N) and (b) SARS-CoV-2 spike (S) proteins, and after blocking with 0.1% Tween-20 and 4% polyethylene glycol.

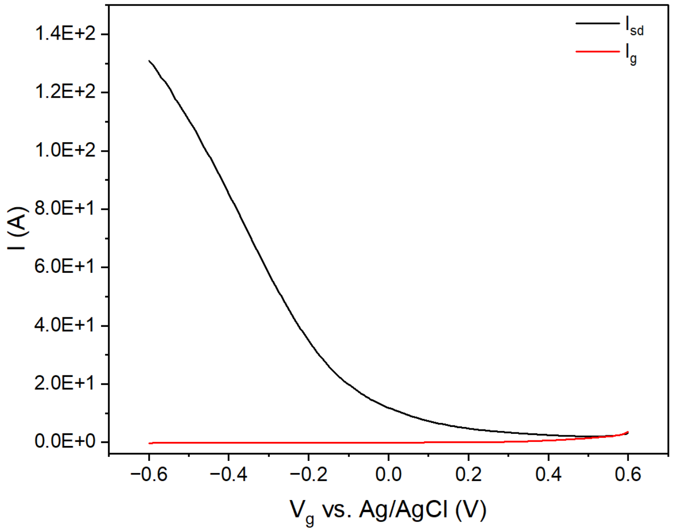

Figure S7. The negligible gate leakage current (I_g) of a HA-SWCNT FET device in comparison to source-drain current (I_{sd}).

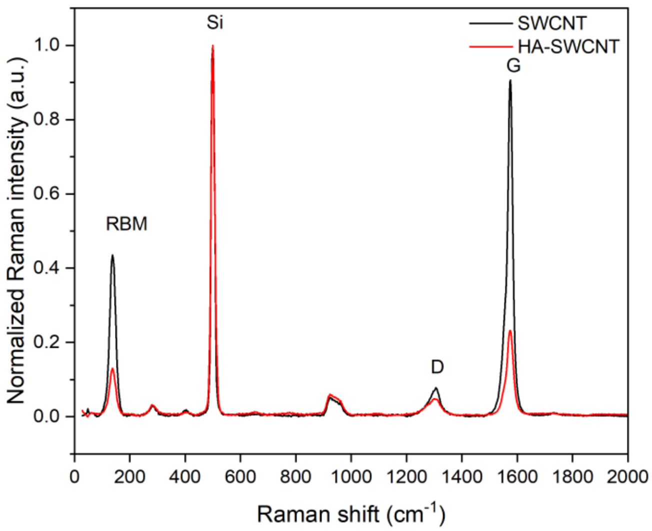

Figure S8. Raman spectra of SWCNTs deposited sensor chips before and after the immobilization of HA antigens. The Raman spectra were recorded using a 638 nm excitation laser.

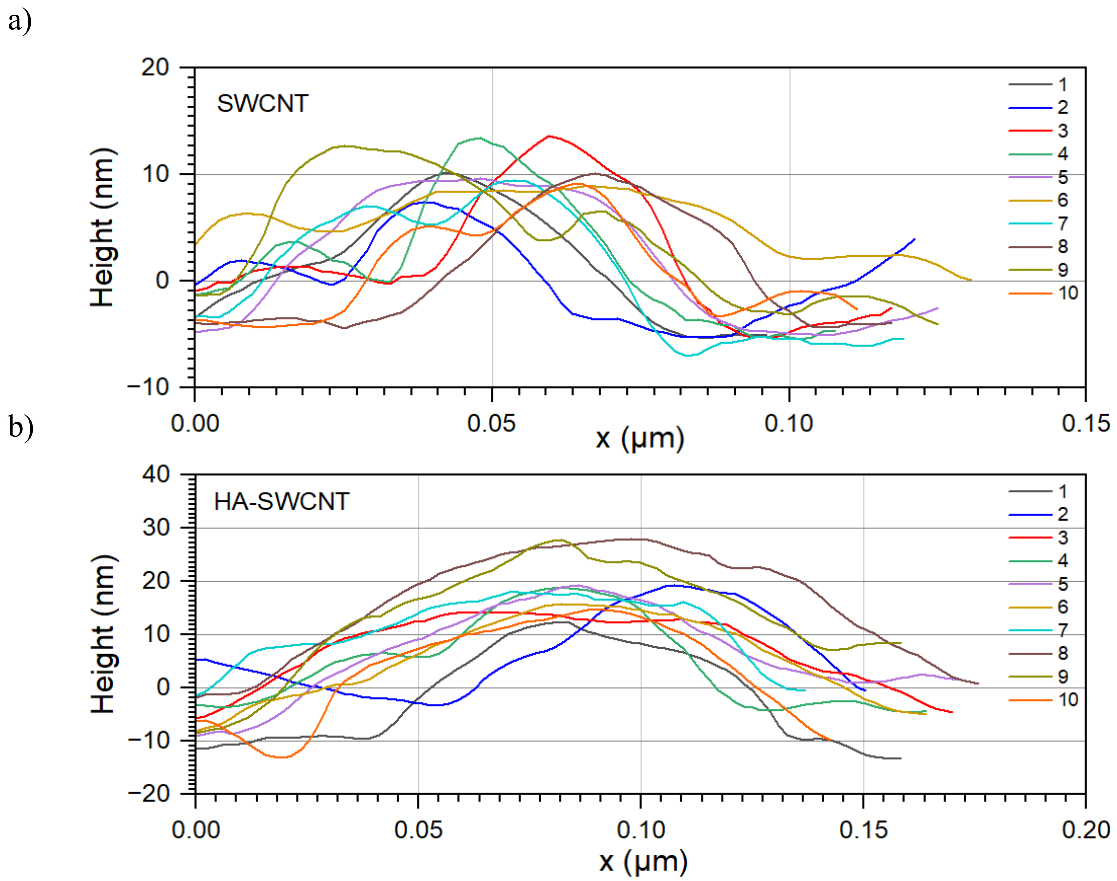

Figure S9. Extracted height profiles from AFM images. Height profiles of (a) SWCNT and (b) HA-SWCNT FET devices.

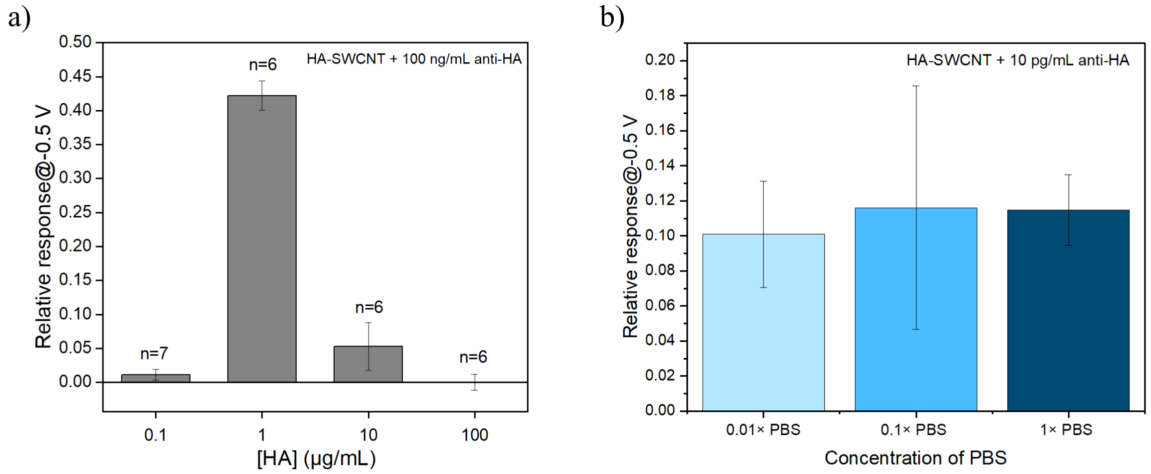

Figure S10. Optimization of HA functionalization and the gate electrolyte. (a) Relative responses after adding 10 μL (100 ng/mL) of anti-HA antibody solutions onto HA-SWCNT FET devices functionalized with different amounts of HA antigens; relative response = $(I–I_0)/I_0$. All plotted data points are mean ± standard error of the mean. The number of devices (n) used for calculations is indicated in the parenthesis in the legend. (b) The effect of gating electrolyte concentration on the FET biosensor relative response to 10 pg/mL anti-HA. All data points plotted in the calibration plots are mean ± standard error of the mean based on 3 devices.

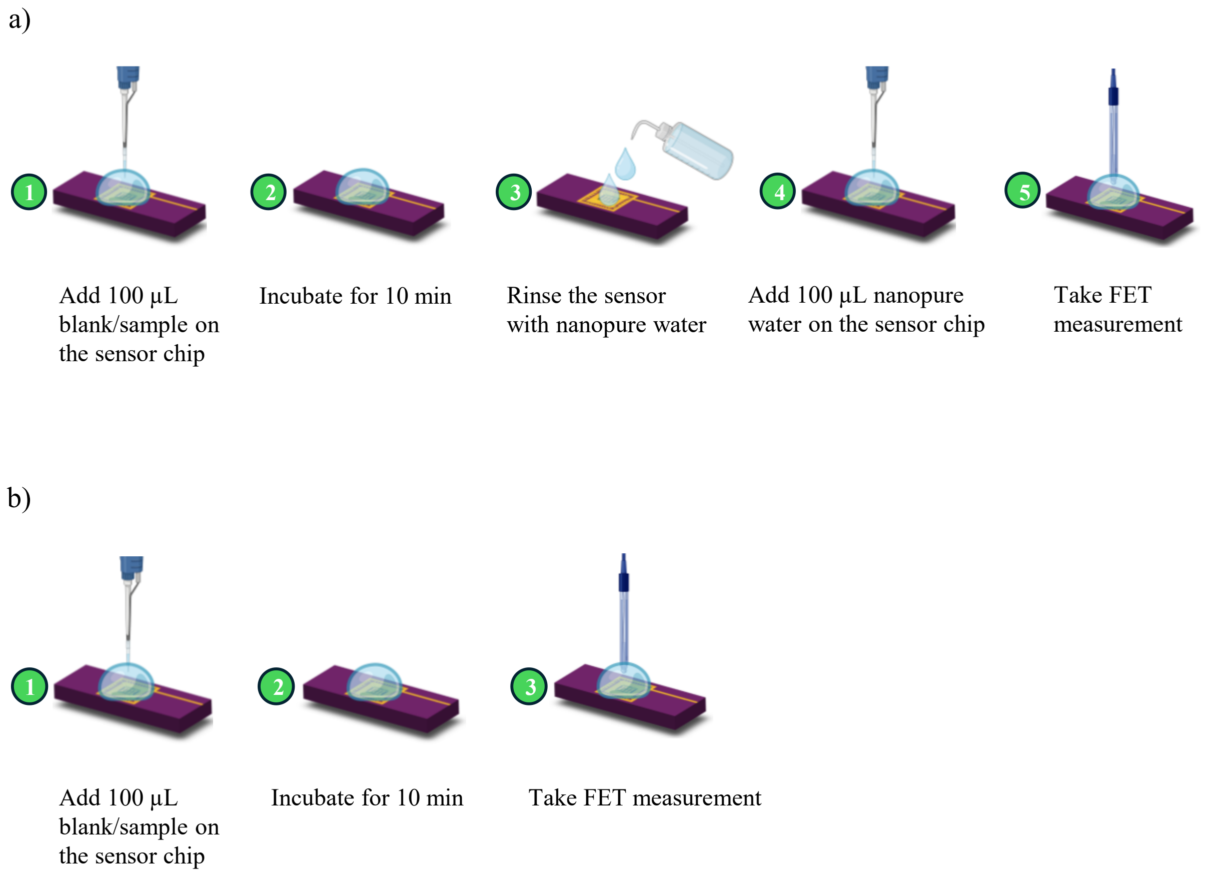

Figure S11. The FET biosensing procedure. (a) Using nanopure water as the gating electrolyte to eliminate Debye length screening effect and increase sensitivity. (b) Direct FET measurements in the sample solution to simplify the sensing procedure.

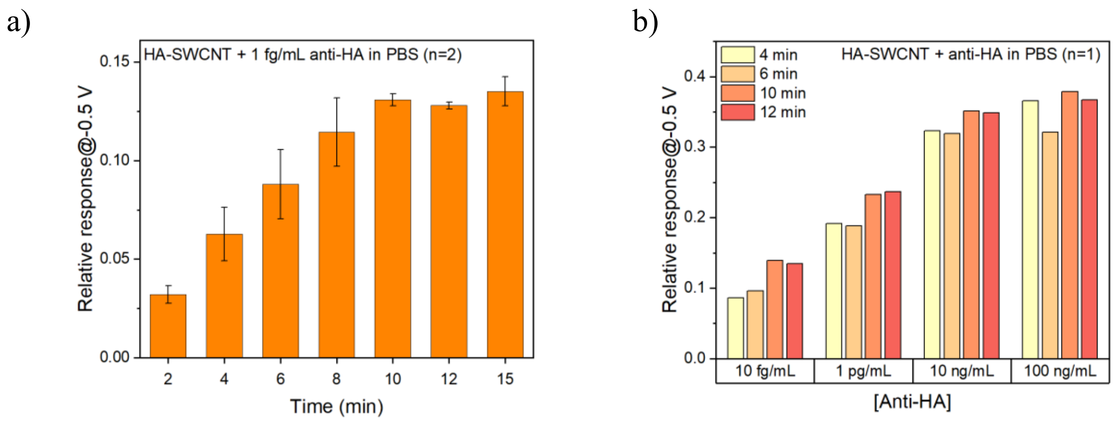

Figure S12. Incubation time optimization. (a) The effect of incubation time on the HA-SWCNT FET biosensor response to 1 fg/mL of anti-HA antibody. (b) The effect of incubation time on the HA-SWCNT FET biosensor response to different concentrations of anti-HA antibody.

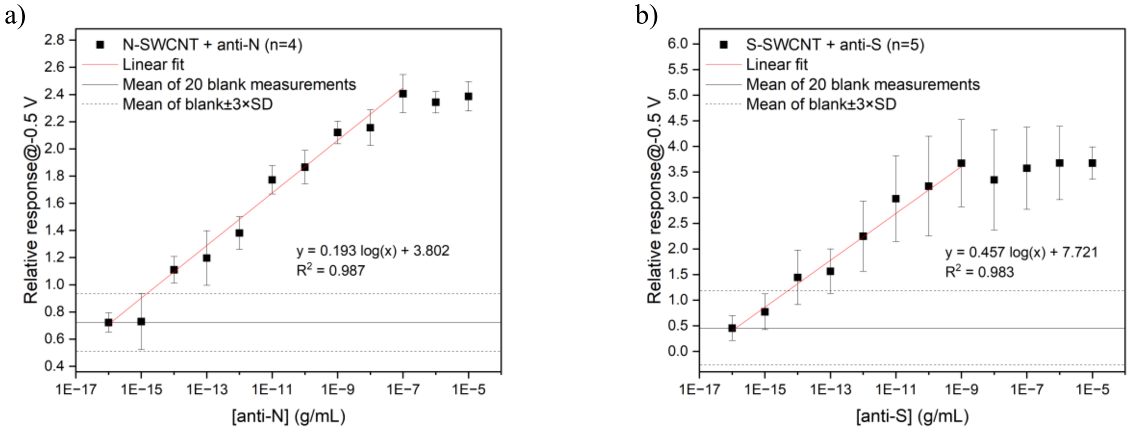

Figure S13. LOD calculations for the detection of anti-SARS-CoV-2 nucleocapsid (anti-N) and anti-SARS-CoV-2 spike (anti-S) antibodies. The LOD was calculated by plugging the smallest sensor response that could be reliably distinguished (x_L) (in this case, the measurements performed with the lowest concentration) into the calibration equations and solving for x value. $x_L$was calculated using the equation $x_L = \bar{x}_B + ks_B$. In this equation, $\bar{x}_B$ represents the mean of the blank measures (in our case, the measurements performed with the lowest concentration), s_B is the standard deviation of the blank measures, and k is set to 3 to achieve a confidence level of 99.6%. For anti-N detection, we found $\bar{x}_B = 0.72242$, $s_B = 0.07109$, and $x_L = 0.93569$, and for anti-S detection, we found $\bar{x}_B = 0.45460$, $s_B = 0.24122$, and $x_L = 1.17826$. The x_L values were plugged into the calibration equations of (a) y = 0.193 log(x) + 3.802 for sensing anti-N and (b) y = 0.457 log(x) + 7.721 for sensing anti-S, and the equations were solved for log(x) equals –14.85135 and –14.31672, respectively. Therefore, LOD was calculated as 1.41 fg/mL ($10^{-14.85135}$ g/mL) for anti-N and 4.82 fg/mL ($10^{-14.31672}$ g/mL) for anti-S.

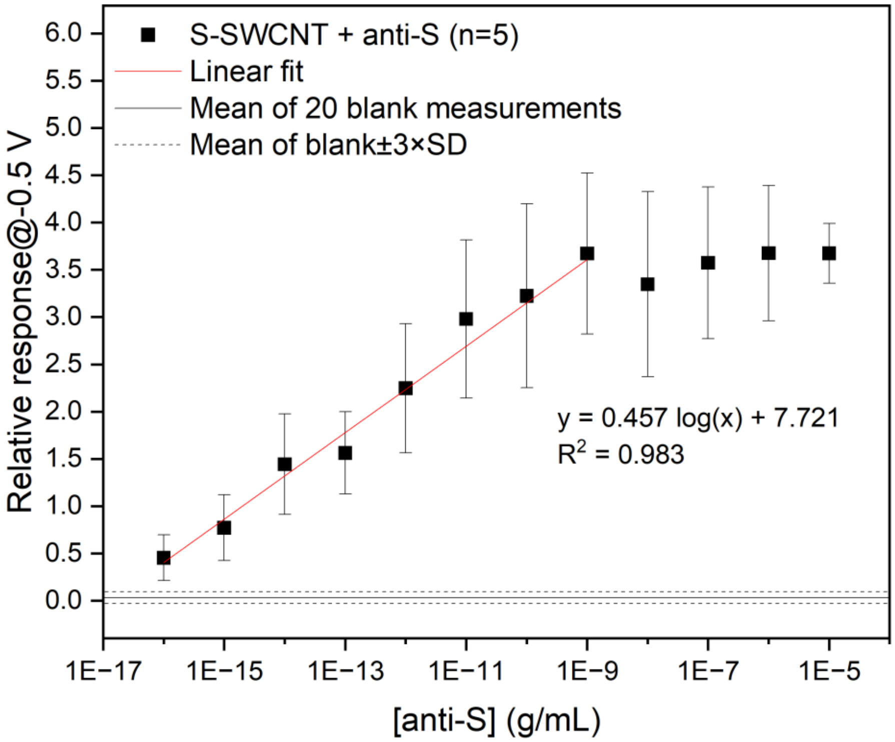

Figure S14. LOD calculations for the detection of anti-SARS-CoV-2 spike (anti-S) antibody. For anti-S detection, we found $\bar{x}_B = 0.03272$, $s_B = 0.02082$, and $x_L = 0.09518$ (in this case, the measurements performed with the blank). The corresponding concentration of anti-S at the calculated sensor response (i.e., the LOD) were then determined by interpolating this value from the fitted calibration curves. The $x_L$value was plugged into the calibration equations of (a) y = 0.457 log(x) + 7.721 for sensing in anti-S and the equations was solved for log(x) equals –16.68670. Therefore, LOD was calculated as 20.6 ag/mL for sensing anti-S antibody.

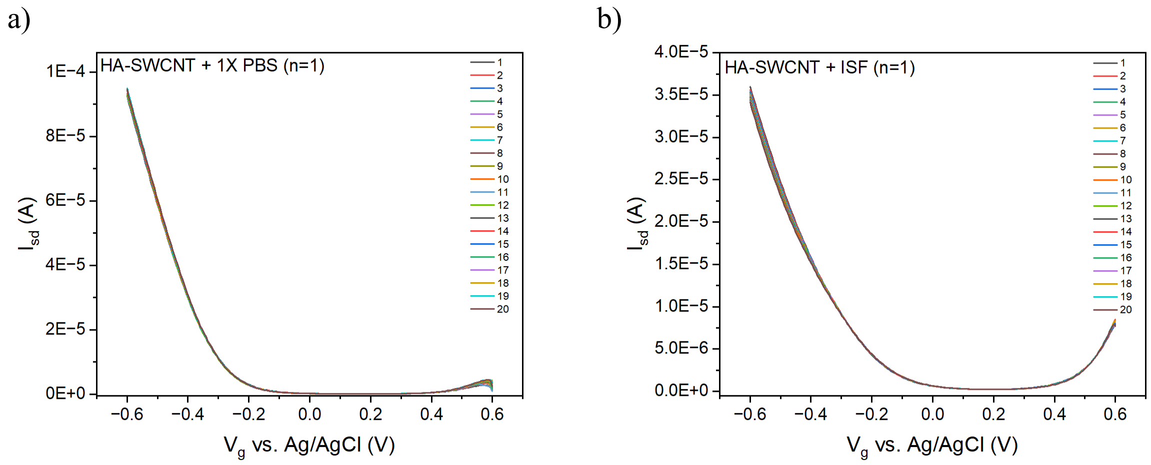

Figure S15. FET characteristics of multiple measurements after 10 min incubation of the HA-SWCNT FET sensor with (a) 100 µL 1✕ PBS and (b) 100 µL artificial ISF.

a) b)

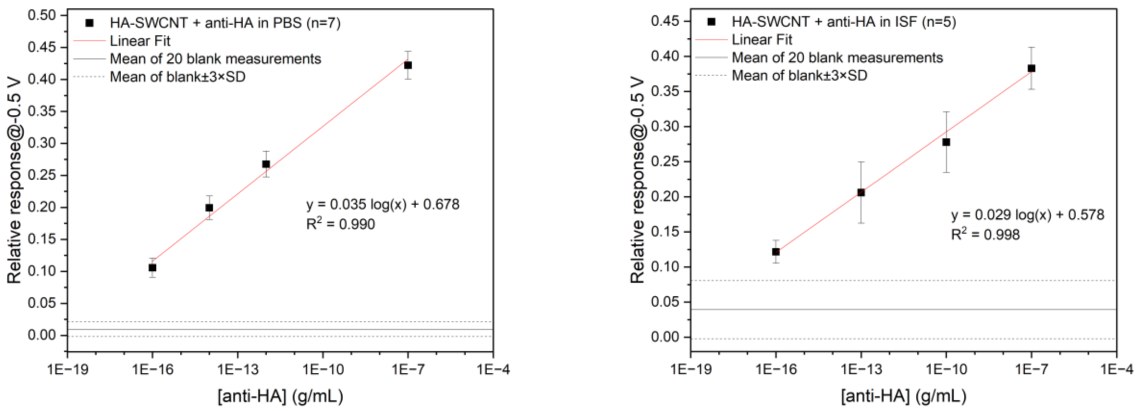

Figure S16. LOD calculations for the detection of anti-HA in 1× PBS and artificial ISF. The calibration curves for the detection of anti-HA antibody in (a) PBS and (b) ISF were linearly fit with linear correlation coefficients of 0.990 and 0.998, respectively. For PBS blank incubation, x_B for 20 times blank measures at –0.5 V was calculated as 0.00944 and s_B was 0.00383. For ISF blank incubation, x_B was calculated as 0.03920 and s_B was 0.01379. So, the range of fluctuation which is $x_L = \bar{x}_B + ks_B$ was calculated as 0.02093 and 0.08057 for 1× PBS and ISF blanks, respectively. The x_L values were plugged into the calibration equations of (a) y = 0.035 log(x) + 0.678 for sensing in 1× PBS and (b) y = 0.029 log(x) + 0.578 for sensing in ISF, and the equations were solved for log(x) equals –18.70664 and –17.15276, respectively. Therefore, LOD was calculated as 0.20 ag/mL ($10^{-18.70664}$ g/mL) for sensing in 1× PBS and 7.0 ag/mL ($10^{-17.15276}$ g/mL) for sensing in ISF. The calibration sensitivity for detection of anti-HA was determined by the slope of the linear region. The calibration sensitivity was 0.035 for sensing in PBS, and 0.029 for sensing in ISF.

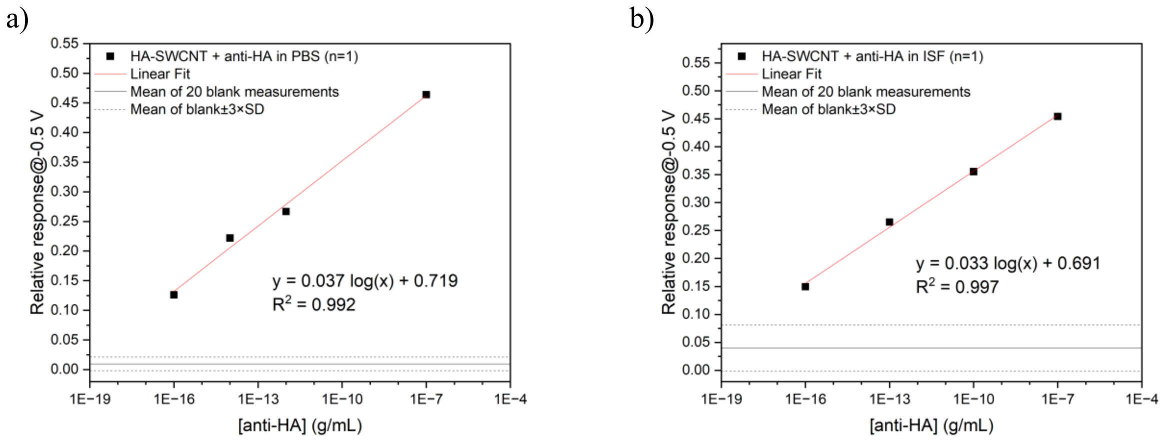

Figure S17. LOD calculations for the detection of anti-HA on a single FET device. For incubating the HA-SWCNT FET device with 1× PBS, x_L was 0.02093 and for incubating the HA-SWCNT FET device with artificial ISF, x_L was 0.08057. The x_L values were plugged into the calibration equations of (a) y = 0.037 log(x) + 0.719 for sensing in 1× PBS and (b) y = 0.033 log(x) + 0.691 for sensing in ISF, and the equations were solved for log(x) equals –19.01553 and –18.49788, respectively. Therefore, LOD was calculated as 0.096 ag/mL for sensing in 1× PBS and 0.32 ag/mL for sensing in ISF.

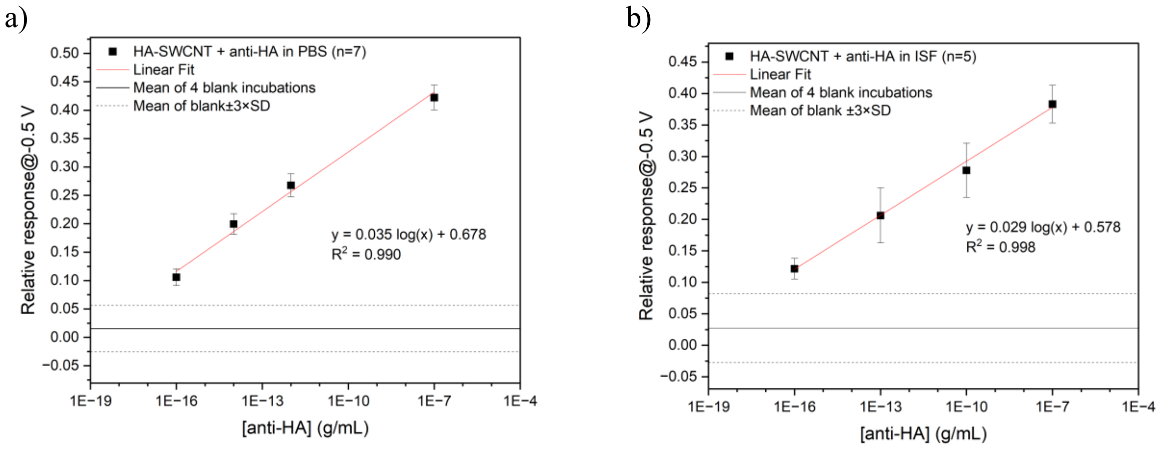

Figure S18. LOD calculations for the applied detection method. For PBS blank incubation, x_B for 4 times blank incubations on HA-SWCNT FET sensor was calculated at –0.5 V as 0.01533 and s_B was 0.01369. For ISF blank incubation, x_B was calculated as 0.02741 and s_B was 0.01824. So, the range of fluctuation which is $x_L = \bar{x}_B + ks_B$ was calculated as 0.05640 and 0.08213 for 1× PBS and ISF blanks, respectively. The x_L values were plugged into the calibration equations of (a) y = 0.035 log(x) + 0.678 for sensing in 1× PBS and (b) y = 0.029 log(x) + 0.578 for sensing in ISF, and the equations were solved for log(x) equals –17.69638 and –17.09897, respectively. Therefore, LOD was calculated as 2.0 ag/mL for sensing in 1× PBS and 8.0 ag/mL for sensing in ISF.

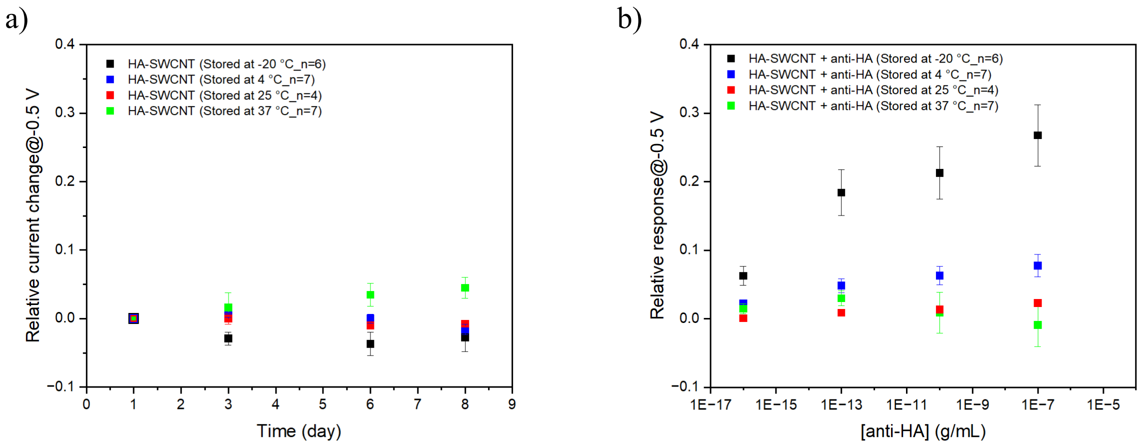

Figure S19. Stability evaluation. (a) The source-drain current change of HA-SWCNT FET biosensors stored under different conditions. (b) Sensing performance of stored biosensors after being exposed to different concentrations of anti-HA antibody.

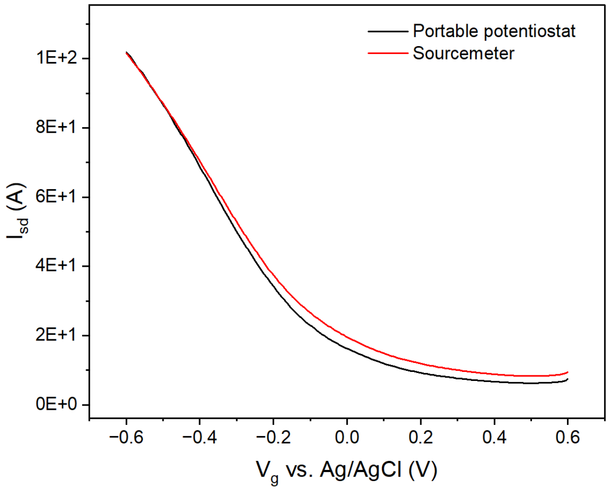

Figure S20. Comparing the portable potentiostat and laboratory high precision sourcemeter in recording FET characteristic curves of a SWCNT FET device.

Table S1. Comparing the proposed biosensor with previously reported techniques.

Method	Target Antibody	Detection Limit	Linear Range	Response Time	Ref.
**ELISA**	anti-SARS-CoV-2 spike	15.0 ng/mL	1 pg/mL – 1 µg/mL	30 minutes	S2
**Surface Plasmon Resonance (SPR)**	anti-SARS-CoV-2 spike	2.7 pg/mL	6.5 ng/mL – 1.6 µg/mL	Several minutes	S3
**Nonradiative energy transfer assay**	anti-SARS-CoV-2 spike	3.0 fg/mL	3.9 pg/mL – 0.5 ng/mL	42 minutes	S4
**Magnetic immuno-detection assay**	anti-SARS-CoV-2 spike	3.4 ng/mL	1.2 ng/mL – 5 µ/mL	42 minutes	S5
**Bioluminescence resonance energy transfer (BRET)**	anti-hemagglutinin	1.1 µg/mL	0.3 µg/mL – 30.0 µg/mL	20 minutes	S6
**Electrochemical**	anti-hemagglutinin	1.0 pg/ml	1 pg/ml – 100 ng/ml	120 minutes	S7
**SWCNT-FET**	anti-SARS-Cov-2 spike	20.6 ag/mL	100 ag/mL – 1 ng/mL	10 minutes	This work
**SWCNT-FET**	anti-hemagglutinin	0.2 ag/mL	100 ag/mL – 100 ng/mL	10 minutes	This work

Table S2. Comparing the SWCNT FET biosensor with other FET-based sensors designed for antibody detection.

FET type	Target Antibody	Detection Limit	Linear Range	Response Time	Ref.
**Silicon Nanowire-FET**	anti-hemagglutinin	0.9 ag/mL	0.1 fg/mL – 1.5 pg/mL	2 minutes	S8
**Triblock Copolymer (TBC)-FET**	anti-SARS-Cov-2 spike	0.4 fg/mL	0.1 fg/mL – 1 μg/mL	20 minutes	S9
**Graphene FET**	anti-SARS-Cov-2 spike	0.4 fg/mL	750 ag/mL – 750 ng/mL	2 minutes	S10
**SWCNT-FET**	anti-SARS-Cov-2 spike	20.6 ag/mL	100 ag/mL – 1 ng/mL	10 minutes	This work
**SWCNT-FET**	anti-hemagglutinin	0.2 ag/mL	100 ag/mL – 100 ng/mL	10 minutes	This work

References

S1. Shao, W.; Burkert, S. C.; White, D. L.; Scott, V. L.; Ding, J.; Li, Z.; Ouyang, J.; Lapointe, F.; Malenfant, P. R. L.; Islam, K.; Star, A., Probing Ca^{2+}-induced conformational change of calmodulin with gold nanoparticle-decorated single-walled carbon nanotube field-effect transistors. *Nanoscale* **2019,** *11* (28), 13397-13406, https://doi.org/10.1039/C9NR03132D.

S2. Krähling, V.; Halwe, S.; Rohde, C.; Becker, D.; Berghöfer, S.; Dahlke, C.; Eickmann, M.; Ercanoglu, M. S.; Gieselmann, L.; Herwig, A.; Kupke, A.; Müller, H.; Neubauer-Rädel, P.; Klein, F.; Keller, C.; Becker, S., Development and characterization of an indirect ELISA to detect SARS-CoV-2 spike protein-specific antibodies. *J Immunol Methods.* **2021,** *490*, 112958, https://doi.org/10.1016/j.jim.2021.112958.

S3. Kausaite-Minkstimiene, A.; Giniunaite, A.; Popov, A.; Ramanaviciene, A., Gold nanoparticle-assisted SPR immunosensor for quantification of SARS-CoV-2 anti-RBD antibodies. *Sens Actuators, B Chem.* **2025,** *432*, 137465, https://doi.org/10.1016/j.snb.2025.137465.

S4. Avila-Huerta, M. D.; Ortiz-Riano, E. J.; Mancera-Zapata, D. L.; Cortes-Sarabia, K.; Morales-Narváez, E., Facile determination of Covid-19 seroconversion via nonradiative energy transfer. *ACS Sens.* **2021,** *6* (6), 2136-2140, https://doi.org/10.1021/acssensors.1c00795.

S5. Pietschmann, J.; Vöpel, N.; Spiegel, H.; Krause, H.-J.; Schröper, F., Brief communication: magnetic immuno-Detection of SARS-CoV-2 specific antibodies. *BioRxiv* **2020**, 2020.06. 02.131102, https://doi.org/10.1101/2020.06.02.131102

S6. Tenda, K.; van Gerven, B.; Arts, R.; Hiruta, Y.; Merkx, M.; Citterio, D., Paper-Based Antibody Detection Devices Using Bioluminescent BRET-Switching Sensor Proteins. *Angewandte Chemie International Edition* **2018,** *57* (47), 15369-15373, https://doi.org/10.1002/anie.201808070.

S7. Arya, S. K.; Kongsuphol, P.; Wong, C. C.; Polla, L. J.; Park, M. K., Label free biosensor for sensitive human influenza virus hemagglutinin specific antibody detection using coiled-coil peptide modified microelectrode array based platform. *Sensors Actuat B-Chem.* **2014,** *194*, 127-133, https://doi.org/10.1016/j.snb.2013.12.066.

S8. Zhang, H.; Osawa, F.; Okamoto, H.; Qiu, Y.; Liu, Z.; Ohshima, N.; Kajisa, T.; Sakata, T.; Izumi, T.; Sone, H., Ultrasensitive Specific Detection of Anti-influenza A H1N1 Hemagglutinin Monoclonal Antibody Using Silicon Nanowire Field Effect Biosensors. *ACS Appl. Bio Mater.* **2025,** *8* (2), 1038-1049, https://doi.org/10.1021/acsabm.4c01263.

S9. Ditte, K.; Nguyen Le, T. A.; Ditzer, O.; Sandoval Bojorquez, D. I.; Chae, S.; Bachmann, M.; Baraban, L.; Lissel, F., Rapid Detection of SARS-CoV-2 Antigens and Antibodies Using OFET Biosensors Based on a Soft and Stretchable Semiconducting Polymer. *ACS Biomater. Sci. Eng.* **2023,** *9* (5), 2140-2147, https://doi.org/10.1021/acsbiomaterials.1c00727.

S10. Kang, H.; Wang, X.; Guo, M.; Dai, C.; Chen, R.; Yang, L.; Wu, Y.; Ying, T.; Zhu, Z.; Wei, D., Ultrasensitive detection of SARS-CoV-2 antibody by graphene field-effect transistors. *Nano Lett.* **2021,** *21* (19), 7897-7904, https://doi.org/10.1021/acs.nanolett.1c00837.
